# Supporting patients to prepare for total knee replacement: Evidence‐, theory‐ and person‐based development of a ‘Virtual Knee School’ digital intervention

**DOI:** 10.1111/hex.13855

**Published:** 2023-08-22

**Authors:** Anna M. Anderson, Gretl A. McHugh, Christine Comer, Judith Joseph, Toby O. Smith, Lucy Yardley, Anthony C. Redmond

**Affiliations:** ^1^ Leeds Institute of Rheumatic and Musculoskeletal Medicine University of Leeds Leeds UK; ^2^ NIHR Leeds Biomedical Research Centre Leeds UK; ^3^ School of Healthcare University of Leeds Leeds UK; ^4^ Musculoskeletal and Rehabilitation Services Leeds Community Healthcare NHS Trust Leeds UK; ^5^ Centre for Clinical and Community Applications of Health Psychology University of Southampton Southampton UK; ^6^ School of Health Sciences University of East Anglia Norwich UK; ^7^ Nuffield Department of Orthopaedics, Rheumatology and Musculoskeletal Sciences University of Oxford Oxford UK; ^8^ School of Psychological Science University of Bristol Bristol UK; ^9^ Present address: Leeds Institute of Health Sciences University of Leeds Leeds UK; ^10^ Present address: Warwick Medical School University of Warwick Warwick UK

**Keywords:** digital intervention, intervention development, mixed methods, prehabilitation, pre‐operative education, total knee replacement

## Abstract

**Introduction:**

Digital delivery of pre‐operative total knee replacement (TKR) education and prehabilitation could improve patient outcomes pre‐ and post‐operatively. Rigorously developing digital interventions is vital to help ensure they achieve their intended outcomes whilst mitigating their potential drawbacks.

**Objective:**

To develop a pre‐operative TKR education and prehabilitation digital intervention, the ‘Virtual Knee School’ (VKS).

**Methods:**

The VKS was developed using an evidence‐, theory‐ and person‐based approach. This involved a mixed methods design with four phases. The first three focused on planning the VKS. The final phase involved creating a VKS prototype and iteratively refining it through concurrent think‐aloud interviews with nine patients who were awaiting/had undergone TKR. Meta‐inferences were generated by integrating findings from all the phases. ISRCTN registration of the overall project was obtained on 24 April 2020 (ISRCTN11759773).

**Results:**

Most participants found the VKS prototype acceptable overall and considered it a valuable resource. Conversely, a minority of participants felt the prototype's digital format or content did not meet their individual needs. Participants' feedback was used to refine the prototype's information architecture, design and content. Two meta‐inferences were generated and recommend:

1. Comprehensive pre‐operative TKR education and prehabilitation support should be rapidly accessible in digital and non‐digital formats.
2. Pre‐operative TKR digital interventions should employ computer‐ and self‐tailoring to account for patients' individual needs and preferences.

**Conclusions:**

Integrating evidence, theory and stakeholders' perspectives enabled the development of a promising VKS digital intervention for patients awaiting TKR. The findings suggest future research evaluating the VKS is warranted and provide recommendations for optimising pre‐operative TKR care.

**Patient or Public Contribution:**

Patient and Public Involvement (PPI) was central throughout the project. For example, PPI representatives contributed to the project planning, were valued members of the Project Advisory Group, had key roles in developing the VKS prototype and helped disseminate the project findings.

## 1 INTRODUCTION

Total knee replacement (TKR) is a transformative operation for many patients with end‐stage knee osteoarthritis.^1^ Correspondingly, the worldwide demand for TKR is high and growing.^1^ Large numbers of patients face lengthy waits for TKR, especially since the start of the coronavirus disease 2019 (COVID‐19) pandemic.^2^ During their wait, patients typically experience severe pain and difficulty with daily activities.^2^ Even after TKR, around 10%–20% of patients report poor outcomes, such as persistent pain or dissatisfaction.^3^, ^4^, ^5^

Numerous predictors of poor post‐TKR outcomes have been identified, including worse pre‐operative pain and function, low musculoskeletal health literacy and unfulfilled outcome expectations.^6^, ^7^, ^8^ Pre‐operative TKR interventions can potentially modify these predictors. Pre‐operative TKR education is particularly important for setting realistic expectations and supporting patients to actively engage in their care.^9^, ^10^ There is also growing evidence that prehabilitation (health/wellbeing optimisation) interventions may improve patient outcomes pre‐ and post‐TKR.^11^, ^12^, ^13^ Despite this, current United Kingdom (UK) National Health Service (NHS) pre‐operative TKR intervention provision is variable, inefficient and often inadequate.^14^, ^15^ When provided, pre‐operative TKR support has traditionally been delivered via face‐to‐face group classes, often called ‘knee schools’.^14^, ^16^ These present various limitations. For example, they are time‐consuming to deliver, and patients may not remember the information provided.^9^, ^17^

Delivering pre‐operative TKR support via a digital intervention could help address these limitations and aligns with the NHS's focus on digital transformation.^18^, ^19^ Preliminary evidence suggests TKR digital interventions may improve patient outcomes and be cost‐effective.^20^ However, digital interventions also have many potential drawbacks. For example, engagement with digital interventions is often low and varies between different patient subgroups; this can limit their effectiveness and risks increasing health inequities.^21^, ^22^ Rigorously developing digital interventions is essential to mitigate these drawbacks. Despite this, a recent review of educational joint replacement digital interventions reported none had been developed using a validated framework or co‐designed with the intended users.^23^ Other reviews have raised concerns about the quality of TKR apps and YouTube videos, and the readability of online TKR educational resources.^24^, ^25^, ^26^

**Figure 1:**
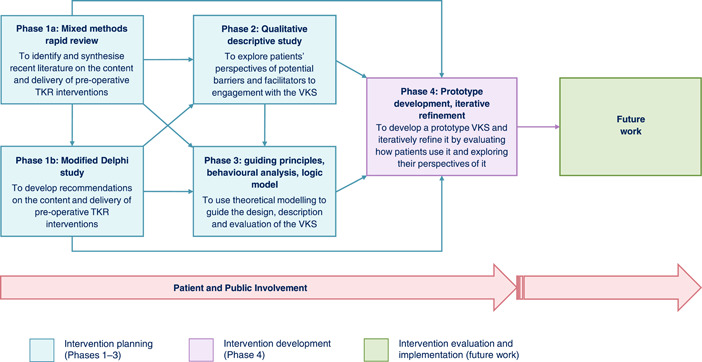
Project flowchart. Flowchart showing the design and objective of each project phase, and how the phases link to each other and future work. Arrows at the bottom of the flow chart demonstrate that Patient and Public Involvement (PPI) was central throughout the project, and PPI is anticipated to be central during any future related work. Pre‐op, pre‐operative; TKR, total knee replacement; VKS, Virtual Knee School.

To help address the above issues, this project aimed to develop a novel UK‐based pre‐operative TKR education and prehabilitation digital intervention, the ‘Virtual Knee School’ (VKS).^27^ Figure 1 provides the objective of each project phase. In line with current guidance,^28^ this paper comprehensively reports the VKS development process. Phases 1 and 2 are reported elsewhere,^29^, ^30^ so this paper focuses on the overall project and Phases 3 and 4.

## 2 METHODS

### 2.1 Design

The VKS was developed using an evidence‐, theory‐ and person‐based approach (PBA). This involved drawing on the Medical Research Council (MRC) framework for developing and evaluating complex interventions^31^ and the PBA.^32^ The MRC framework was chosen because it provides well‐established, flexible guidance.^31^ The PBA was identified as a valuable complementary approach as it provides more detailed guidance on intervention development actions.^32^ Furthermore, it focuses on understanding the intended users' psychosocial contexts and perspectives, with the aim of ensuring interventions are acceptable and engaging for users.^32^ This was considered particularly important because the VKS was designed to be used without health professional support. The PBA's core elements include iterative qualitative/mixed methods research and the creation of ‘guiding principles’ (summary of the key intervention design objectives and features).^32^, ^33^

A mixed methods design with four phases was employed (Figure 1). The design was considered using the typology of Creswell et al.,^34^ who describe three ‘core’ mixed methods designs. These can be used in isolation or intersected with other approaches to form a ‘complex’ design. This project's design was considered complex as it involved multiple phases and each empirical phase had a qualitative or mixed methods design. The project's intent most closely aligns with that of an ‘exploratory sequential’ core design, which involves using qualitative data to inform a novel ‘quantitative feature’ (e.g., an intervention or instrument), which is then quantitatively evaluated. A process evaluation of the VKS was initially planned but could not be conducted due to factors such as the COVID‐19 pandemic and the large volume of content included in the VKS. The project's overarching design was still considered exploratory sequential, which helped to ensure that validity concerns associated with the exploratory sequential design were identified and addressed. For example, to demonstrate that the qualitative/mixed methods findings informed the quantitative feature, the findings of the intervention planning phases were explicitly linked to the VKS features.

Each phase was given equal priority, conducted largely sequentially, and informed by the preceding phase(s). Correspondingly, all the phases involved integration through building.^35^ Additionally, all the phases' findings were integrated to generate ‘meta‐inferences’. Meta‐inferences provide a more complete understanding of a topic than the inferences of the separate strands of a mixed methods study, as they are overall conclusions developed by integrating findings from the separate strands.^36^ Generating the meta‐inferences involved linking inferences from the different phases; assessing potential similarities/differences; developing credible explanations of the similarities/differences; and identifying implications of the findings.^36^ Consistent with the mixed methods design, the project was primarily underpinned by pragmatism.^34^

### 2.2 Ethical approval, registration and reporting

Ethical approval for Phase 1b and Phases 2–4 was gained from the London – Riverside Research Ethics Committee (REC) (19/LO/0813) and Yorkshire and The Humber – Bradford Leeds REC (20/YH/0095) respectively. All participants provided electronic informed consent before participating. ISRCTN registration of the overall project was obtained on 24 April 2020 (ISRCTN11759773). The primary reporting guideline used for this paper was the GUIDance for the rEporting of intervention Development (GUIDED) checklist.^28^

### 2.3 Research team and oversight

This project was led by a female physiotherapist (A.M.A.) during her full‐time Clinical Doctoral Research Fellowship. Before the project, she had gained relevant skills through her clinical/academic work (e.g., interviewing skills) but had not conducted an intervention development project. Other team members have expertise in multiple relevant areas including orthopaedics, qualitative/mixed methods research and digital behaviour change intervention development.

The project was overseen by a Project Advisory Group (PAG), which included an independent chair, a local collaborator, the lead researcher, three of her supervisors and three Patient and Public Involvement (PPI) representatives (one of whom was recruited halfway through the project to improve the group's ethnic diversity). The PAG contributed to multiple activities such as defining the project success criteria and monitoring the project progress. The PAG met approximately every 6 months throughout the 39‐month project. Additional meetings with specific members were organised when required.

### 2.4 PPI

PPI was central throughout the project with the aim of ensuring the research procedures and VKS were acceptable and inclusive, and the findings were effectively disseminated (Table 1).

**Table 1:** PPI overview.

Activity	Description	Example of impact
Project planning consultations	Seven NIHR Leeds BRC PPI representatives joined a general consultation, which involved discussing pre‐operative TKR care. Two NIHR Leeds BRC PPI representatives joined a more focused consultation, which involved reviewing an existing digital intervention for people with joint pain.	The VKS was developed as a website (rather than a mobile application) to help maximise accessibility.
PAG meetings and follow‐up	Three PAG PPI members provided oversight of the project by attending PAG meetings and contributing to follow‐up activities as required.	The phase 4 recruitment procedures were amended to include a PAG PPI member sharing a WhatsApp recruitment message with contacts in her communities.
Reviewing documents	Two PAG PPI members and seven NIHR Leeds BRC PPI representatives reviewed the Phase 1b participant documents. Three PAG PPI members reviewed the Phase 2 and/or 4 participant documents and topic guides.	Bold 18 pt text was added to the top of the Phase 2 and 4 Participant Information Sheets to explain how to request the document in large print.
Survey pilot testing	Two PAG PPI members and one additional PPI representative pilot tested the Round 1 survey in the Phase 1b Delphi study.	Explanations of the roles of different health professional teams were added.
Consultations on the VKS content and exercise programme	Two PAG PPI members participated in consultations about the VKS content and exercise programme design.	Extra details were added to the educational video transcripts, for example, about sleep difficulties and psychological wellbeing.
Consultations and coproduction activities during the VKS prototype design, build and testing	Three PAG PPI members contributed to creating a provisional VKS template and style guide; creating the VKS designs; informing the VKS prototype build; and/or formal UAT. Two additional PPI representatives contributed to informal UAT.	Instructions on how to use the accessibility toolbar were added to the ‘About the Virtual Knee School’ and ‘Help’ webpages.
Filming to create VKS videos	Eight volunteer Patient Models were filmed to create the VKS education and exercise videos.	The VKS videos were positively evaluated by participants in Phase 4.
Dissemination of the project findings	Three PAG PPI members reviewed plain English summaries and an infographic of the project findings. Two PAG PPI members contributed to a public dissemination event, which included helping to plan the event and presenting at the event.	PPI input was weaved throughout the dissemination event presentation, rather than being limited to a section on PPI.

Abbreviations: BRC, Biomedical Research Centre; NIHR, National Institute for Health and Care Research; PAG, Project Advisory Group; PPI, Patient and Public Involvement; UAT, user acceptance testing; VKS, Virtual Knee School.

### 2.5 VKS prototype planning (Phases 1–3)

Three intervention planning phases were conducted (Figure 1). Phases 1 and 2 are reported elsewhere,^29^, ^30^ so are not detailed here.

#### 2.5.1 Theoretical modelling (Phase 3)

Phase 3 involved using the following three theoretical modelling approaches to help guide the VKS design, description and evaluation:

1. Creating guiding principles.
2. Undertaking a behavioural analysis.
3. Developing a logic model.

All three approaches were implemented by the lead researcher. The findings were then refined through discussions with other research team members. Further details of each theoretical modelling approach are provided below:

1. *Creating guiding principles*.

In line with the PBA,^32^ guiding principles were created with the aim of ensuring the VKS has a coherent focus and is acceptable and engaging for users. This involved specifying what the VKS aims to provide (outcome objective) and the behaviours it seeks to change (behavioural objective). The objectives were primarily based on the project planning PPI consultations and pre‐operative TKR intervention literature. Next, groups of considerations related to the intended VKS users' characteristics, contexts and needs were identified from the project planning PPI consultations and Phase 1 and 2 findings. Considerations that could not be addressed through a fully automated digital intervention (e.g., direct social support) were excluded. Each group of considerations was used to develop a VKS guiding principle. The VKS guiding principles were designed to be complementary to the PBA common guiding principles (principles proposed to optimise engagement with most digital behaviour change interventions).^32^

2. *Undertaking a behavioural analysis*.

A behavioural analysis was undertaken to systematically analyse each behaviour targeted by the VKS, code potential VKS features using standardised terminology and map the features to the project planning PPI consultations and Phase 1 and 2 findings. The behavioural analysis was conducted using the Behaviour Change Wheel (BCW), a theoretical framework underpinned by the Capability, Opportunity, Motivation, Behaviour (COM‐B) model of behaviour.^37^ The BCW was primarily chosen because it is comprehensive, relatively simple and well‐established; addresses context; and is linked to the Behaviour Change Technique Taxonomy v1 (BCTTv1), a well‐established taxonomy of 93 behaviour change techniques (BCTs).^38^

The behavioural analysis methods were based on previous relevant studies.^39^, ^40^, ^41^ First, behavioural analysis tables were created for each of the VKS's target behaviours. To populate the tables, sets of barriers and facilitators to the target behaviour and potential VKS features that could address the barriers and facilitators were identified from the project planning PPI consultations and Phase 1 and 2 findings. Extra features were added based on research team discussions. All the features were mapped to COM‐B model components, BCW intervention functions and BCTTv1 BCTs.

To check for additional potentially important behavioural targets/intervention components, the behavioural analysis tables were compared to the BCW, BCTTv1 and BCTs identified in a systematic review of digital‐based osteoarthritis self‐management programmes by Safari et al.^42^ The behavioural analysis tables were also compared with the VKS guiding principles to check for inconsistencies.

3. *Developing a logic model*.

A process‐orientated logic model was developed to provide a diagrammatic representation of the VKS, including its proposed causal mechanisms and intended outcomes.^43^ This process was informed by the MRC process evaluation guidance,^44^ other digital intervention logic models^39^, ^40^, ^41^ and Type 4 logic model guidance.^45^ The logic model content was based on the project planning PPI consultations, Phase 1 and 2 findings, additional Phase 3 findings, pre‐operative TKR intervention literature and digital intervention literature.

### 2.6 VKS prototype development and refinement (Phase 4)

Phase 4 involved developing a VKS prototype and iteratively refining it by evaluating how patients used it and exploring their perspectives of it.

#### 2.6.1 VKS prototype development

##### Intervention features selection

In line with PBA guidance,^46^ intervention planning tables were created to collate potential VKS features and document the rationale and priority of each feature. The features were prioritised using the ‘Must have, Should have, Could have, Would like’ (MoSCoW) model^47^, ^48^ based on criteria developed by the research team (Table 2). Separate intervention planning tables were created for each proposed VKS section (Supporting Information: File 1).

**Table 2:** Prioritisation criteria for including features in the VKS prototype.

Code	Reason for inclusion^a^	Importance level	Time‐consuming to develop^b^	Priority^c^
FN	Important for the VKS functioning/navigation.	1	No	Must have
S	Required for safety purposes.		Yes	Must have
R	Required to meet relevant regulations/guidelines.			
VGP (VGP number)	Required to meet one or more VGPs developed in Phase 3.			
CGP (CGP number)	Required to meet one or more person‐based approach CGPs.^32^	2	No	Should have
PPI	Addresses PAG PPI member feedback.		Yes	Could have
PAS	Addresses BSI PAS 277:2015 quality criteria.^49^			
NICE	Addresses the NICE primary joint replacement guideline.^14^			
VIR (item number)	Addresses one or more items prioritised as ‘Very important’ in the Phase 1b modified Delphi study final recommendations.^30^			
IR (item number)	Addresses one or more items prioritised as ‘Important’ in the Phase 1b modified Delphi study recommendations.^30^	3	No	Could have
			Yes	Would like
BF (barrier/facilitator set^d^)	Addresses one or more barriers/facilitators identified in the Phase 3 behavioural analysis.			

Abbreviations: BSI, British Standards Institution; CGP, common guiding principle; NICE, National Institute for Health and Care Excellence; PAG, Project Advisory Group; PAS, publicly available specification; PDF, portable document format; PPI, Patient and Public Involvement; VGP, Virtual Knee School guiding principle; VKS, Virtual Knee School.

^a^ Key findings from the Phase 1a rapid review,^29^ Phase 1b modified Delphi study free‐text comments,^30^ and Phase 2 qualitative descriptive study were covered by the modified Delphi study recommendations and behavioural analysis; therefore, they were not listed as reasons for inclusion to help keep the length/complexity of the table manageable.

^b^ Features were classed as time‐consuming to develop if they would require substantial programming time or involve developing a video, photograph, infographic or PDF document.

^c^ If a feature was supported by more than one reason, the priority was based on the reason with the highest importance level.

^d^ The barrier/facilitator sets were labelled with the codes reported in the behavioural analysis tables (Supporting Information: File 2).

All ‘Must have’ and ‘Should have’ features, some ‘Could have’ features and no ‘Would like’ features were included. The selection of ‘Could have’ features was primarily based on consensus within the research team on how important each feature was perceived to be and the time required to develop it.

##### Content development

The content was drafted by the lead researcher and informed by research team discussions and PAG PPI member consultations. Sources used to inform the content included the Phase 1–3 findings, additional relevant research, other digital interventions research team members had helped develop, relevant guidelines,^14^, ^49^, ^50^, ^51^, ^52^, ^53^, ^54^, ^55^ publicly available information from respected sources^56^, ^57^, ^58^, ^59^, ^60^, ^61^, ^62^ and West Yorkshire Association of Acute Trusts orthopaedic education resources. The exercise programme was designed using a multistep process (Supporting Information: File 1). Key priorities during the content development included addressing the VKS and common guiding principles and promoting accessibility/inclusion.

##### Prototype design, build and testing

A web design/development company called ‘Frank’ was commissioned to create and host the VKS prototype.^63^ This involved a multistage design, build and testing process informed by Frank's well‐established procedures (Table 3).

**Table 3:** VKS prototype design, build and testing process.

Stage	Details	Activities^a^	Contributors^b^	Impact of feedback (key points)
Creation of a provisional VKS template and style guide	The lead researcher drafted six potential VKS design templates in Microsoft PowerPoint 2016, then used feedback on these to create a provisional VKS template and style guide.	Online Project Advisory Group meeting Online research team meeting One additional online meeting Telephone call Email correspondence	Two PAG PPI members PAG key collaborator member Four research team members	A turquoise/purple/blue colour scheme and a banner with three coloured triangles were chosen, as they were perceived to be the most aesthetically pleasing. A logo of a person demonstrating the knee straightening exercise was included without a motto, as the motto text would have been very small.
Creation of the VKS designs	The Frank team used the provisional VKS template, style guide and content documents/files to create VKS designs in a PDF document, and then iteratively refined them based on the feedback obtained.	Two online scoping coproduction sessions with a member of the Frank team Two additional online meetings Telephone call	Three PAG PPI members PAG independent chair Three research team members	Instructions on how to use the accessibility toolbar were added to the *‘*About the Virtual Knee School’ and ‘Help’ pages due to concerns that users may miss the toolbar and/or not know how to use it. The ‘slider’ (rotating content in the website banner) proposed by the Frank team was removed due to concerns about its accessibility. ‘Your most viewed pages’ hyperlinks were added to the footer to enable users to quickly navigate to their most frequently viewed pages.
Build of the VKS prototype	The Frank team used the refined designs and content documents/files to build the VKS prototype on their Content Management System, and then iteratively refined it based on the feedback obtained.	Two online show and tell coproduction sessions with two members of the Frank team	One PAG PPI member Four research team members	The instructions on how to use the accessibility toolbar were moved from the bottom to the top of the ‘About the Virtual Knee School’ page to make them more obvious. Extra colour was added to the goal‐setting page and icons were added to the goal‐review page to make the pages more visually appealing. The goal review time limit was removed to allow users to review their goals at any time rather than needing to wait a week to maximise flexibility.
User acceptance testing	The lead researcher provided each formal tester with individualised instructions for testing the VKS prototype. The instructions were designed to ensure that all key functions were tested using a range of devices, operating systems, browsers, and accessibility software. Informal testers were invited to view the prototype and provide general comments. The lead researcher collated the feedback in a test log and addressed the feedback herself where possible and asked the Frank team to address it if not.	Two online testing sessions One face‐to‐face testing session Online research team meeting Email correspondence	Formal testers: Three PAG PPI members Five research team members Informal testers: Two PPI representatives PAG key collaborator Four health professionals/researchers	Navigation instructions were added to the ‘About the Virtual Knee School’ and ‘Help’ pages for clarity. The word ‘surgery’ was changed to ‘operation’ where appropriate to improve clarity and readability, particularly for people with English as an additional language. *‘*Video’ was added to the titles of the videos to make it clear they were videos not static images. Instructions on how to play the videos/change the video settings were added as accordion content (expandable headings) to all videos for clarity. Captions were turned on by default on all videos to improve accessibility. The login process and goal‐setting feature error messages were updated for clarity. Back and next buttons were labelled with the names of the pages they go to for clarity. Buttons were added to the final page in each section to allow users to return directly to the homepage to improve the ease of navigation.

Abbreviations: PAG, Project Advisory Group; PDF, portable document format; PPI, Patient and Public Involvement; VKS, Virtual Knee School.

^a^ The term ‘coproduction’ refers to activities in which PAG PPI members played a direct role in making decisions.^64^

^b^ The lead researcher was involved in all the stages but is not listed in the contributor's column.

#### 2.6.2 VKS prototype evaluation and refinement

##### Overview

A think‐aloud study was undertaken to evaluate the VKS prototype's usability, explore patients' perspectives of it, and prioritise and implement changes to it. The think‐aloud method was chosen because it allows users' immediate responses to an intervention to be observed/explored, enabling important content and navigational issues to be addressed before evaluating the intervention in real‐world settings.^32^, ^47^ Multiple strategies were employed to ensure trustworthiness. For example, an audit trail was maintained and the lead researcher kept a reflexive journal.

##### Participants

Patients were recruited from an NHS teaching hospital by posting recruitment packs to patients and discussing the study with patients at orthopaedic and preassessment clinics. Patients who heard about the study via word of mouth were also included. Additional recruitment activities were employed with the aim of facilitating the recruitment of patients who were male and/or from a Black, Asian or other minority ethnic group (Supporting Information: File 1). None of the additional activities led to the recruitment of any participants.

Adults able to give informed consent were eligible for inclusion if they were:

- able to communicate in English;
- listed for primary TKR at a UK hospital and/or had undergone primary TKR at a UK hospital within the past two years; and
- able to use and had access to the Internet and email and/or were willing and able to be interviewed in person.

To help ensure the VKS meets the needs of diverse patients, maximum variation purposive sampling was employed based on age, gender, ethnicity, highest educational qualification completed, varying experience of TKR and varying confidence in using the Internet.^65^

Nine participants were interviewed as analysis of the eighth and ninth participants' interviews did not suggest any substantial changes should be made to the VKS prototype, suggesting the sample size was sufficient.^66^ Seven participants were patients at the hospital where the lead researcher was based, but none had received care from the lead researcher or any other research team member before the study.

##### Data collection

The lead researcher undertook the data collection independently between 13 October 2021 and 20 January 2022. All participants were invited to participate in two concurrent think‐aloud interviews.^67^, ^68^ To meet COVID‐19 guidance, participants were encouraged to participate remotely via Microsoft Teams but could participate in person if necessary (e.g., if they lacked internet access). All four participants who requested in‐person interviews chose to be interviewed in their own homes. All the participants were aware that the lead researcher's Ph.D. was focused on developing the VKS, which may have encouraged them to provide socially desirable feedback.^66^ To help address this, the researcher emphasised that negative comments would be particularly valuable for refining the VKS prototype.

Each interview was guided by a topic guide (Supporting Information: File 1). To ensure that sufficiently detailed information was obtained, an interactive think‐aloud interview style was employed.^69^, ^70^ This involved the researcher instructing the participant to work through the VKS prototype whilst speaking their thoughts out loud, asking them probing questions, and directing them to specific pages/aspects when appropriate. Additionally, the researcher asked brief semi‐structured questions towards the end of each interview to explore the participant's perspectives of the prototype overall. To the lead researcher's knowledge, all the participants were alone during their interviews. One participant's health problems made it difficult/painful for her to use a digital device, so the lead researcher performed the manual actions required to navigate the prototype for this participant in line with her directions.

All the interviews were video‐ and audio‐recorded except for one in‐person interview, which was not video‐recorded due to an error. The lead researcher documented field notes during and/or shortly after each interview. Interviews lasted between 23 and 87 min (median: 63 min; interquartile range: 17 min) and were transcribed intelligent verbatim by a professional transcription company.

##### Data analysis

Data were analysed using the approach described by Bradbury et al.,^66^ which facilitates efficient systematic analysis of qualitative data during intervention refinement studies. This involved the lead researcher working through each transcript line by line to identify positive and negative comments about the VKS prototype. Changes that could be made to address each negative comment were identified and prioritised. The prioritisation was undertaken using the MoSCoW model^47^, ^48^ based on criteria adapted from Bradbury et al.^66^ and the other PBA resources^71^, ^72^ (Table 4). The research team discussed the potential changes and agreed on which changes to implement.

**Table 4:** Criteria for implementing changes to the VKS prototype.

Code	Reason for change^a^	Importance level	Time consuming to implement^b^	Priority^c^
FSR	Important for the VKS functioning/navigation, safety or compliance with relevant regulations/guidelines.	1	No	Must have
			Yes	Must have
VGP (VGP number)	Consistent with the VGPs developed in Phase 3.			
CGP (CGP number)	Consistent with the person‐based approach CGPs.^32^	2	No	Should have
EEQ (type)	Consistent with experience, evidence and/or the BSI PAS 277:2015 quality criteria.^49^ This includes changes supported by PAG member feedback, the NICE primary joint replacement guideline,^14^ the Phase 1b modified Delphi study recommendations^30^ and/or the expertise of the research team.		Yes	Could have
BEH (target behaviour)	Likely to impact engagement with any of the following:			
	pre‐op TKR care in a web‐based format; pre‐op TKR education; a pre‐op TKR exercise programme; healthy lifestyle changes.			
	This includes, but is not limited to, changes that address barriers/facilitators identified in the Phase 3 behavioural analysis and changes that impact precursors to the desired behaviours, for example, acceptability, accessibility, persuasiveness and so on.			
REP	Addresses a point repeated by more than one participant.			
EAS	Easy and uncontroversial as it does not require any substantial design changes, for example, amending a sentence for clarity.	3	No	Could have
			Yes	Would like
NTC	Does not contradict any of the criteria listed above. (Only listed in the table of changes if none of the criteria above applied.)			
NTA (reason)	Not appropriate, for example, due to contradicting one of the criteria listed above.	N/A	N/A	N/A

Abbreviations: BSI, British Standards Institution; CGP, common guiding principle; N/A, not applicable; NICE, National Institute for Health and Care Excellence; PAG, Project Advisory Group; PAS, publicly available specification; PDF, portable document format; PPI, Patient and Public Involvement; pre‐op, pre‐operative; TKR, total knee replacement; VGP, Virtual Knee School guiding principle; VKS, Virtual Knee School.

^a^ Reasons for change criteria adapted from Bradbury et al.^66^ and additional person‐based approach resources.^71^, ^72^

^b^ Changes were classed as time‐consuming to implement if they required substantial programming time; involved amending multiple pages; involved amending a static image or video; and/or involved developing a new page, video, photograph, infographic or PDF document.

^c^ If a change was supported by more than one reason, the priority was based on the reason with the highest importance level. Changes considered ‘Not appropriate’ were not prioritised.

The analysis was documented in a ‘table of changes’ in Microsoft Excel^73^ (Supporting Information: File 1). Comments were also coded using QSR International NVivo software (Version 12 and Release 1) to ensure verbatim comments were readily accessible.

The data analysis and implementation of changes were conducted concurrently with the data collection to enable the impact of changes made based on earlier interviews to be explored in subsequent interviews.^32^ Member checking was not employed due to the rapid iterative nature of the analysis.

## 3 RESULTS

### 3.1 VKS prototype planning (Phases 1–3)

The Phase 1–2 findings are reported elsewhere,^29^, ^30^ so only the Phase 3 findings are detailed below.

#### 3.1.1 Theoretical modelling (Phase 3)

1. *Creating guiding principles*.

The following VKS objectives were specified.

- *Outcome objective*: to provide a patient‐centred, widely accessible and cost‐effective pre‐operative TKR education and prehabilitation resource.
- *Behavioural objective*: to support patients listed for primary TKR to engage with pre‐operative TKR care in a web‐based format, pre‐operative TKR education, a pre‐operative TKR exercise programme and healthy lifestyle changes.

Six groups of considerations related to the intended VKS users' characteristics, context and needs were identified (Supporting Information: File 2), each of which informed a VKS guiding principle (Table 5).

**Table 5:** VKS guiding principles.

VGP	Intervention design objective	Key intervention features
1	To provide a cost‐effective, credible source of pre‐operative TKR education and prehabilitation support that is widely/immediately accessible, easy to use and engaging for a wide range of users.	Being fully automated.
		Emphasising that the VKS is evidence‐based, developed by a team of UK‐based experts, and linked to the NHS.
		Ensuring all sections can be accessed rapidly during any session.
		Providing clear instructions on how to use the VKS, including a ‘Help’ page at a minimum.
		Ensuring that the navigation and features are simple and quick to use.
		Providing PDF versions of key content/digital tools that users can download and print out, including a PDF exercise booklet and the documents listed under VGP‐4 at a minimum.
2	To address users' potential concerns about pre‐operative TKR education.	Emphasising that the VKS does not include any graphic details of TKR surgery.
		Providing brief information about TKR surgery only, without any graphic details.
		Ensuring that a range of appropriately moderated patient stories is provided, which are unlikely to be interpreted as ‘horror stories’, and highlighting that everyone's preparations for/recovery from TKR surgery are different.
3	To account for users' varying pre‐operative TKR education preferences and needs.	Providing pre‐operative TKR education in accessible and engaging formats, ensuring key information is kept brief, but more detailed information is available for users who wish to access it.
		Providing information using simple language, avoiding medical terms where possible.
		Providing a glossary of medical terms.
		Providing key information using pictures and videos where appropriate, including videos related to understanding what to expect, pain management and rehabilitation (including using walking aids) at a minimum.
4	To address users' potential misconceptions about pre‐operative TKR exercise and build their motivation to engage with the VKS exercise programme.	Providing reassurance that performing pre‐operative exercises is safe for people with severe knee arthritis.
		Explaining the potential benefits of performing pre‐operative exercises, including for post‐operative recovery.
		Including patient stories modelling how other patients have benefitted from performing pre‐operative TKR exercises.
		Providing features designed to motivate users to engage with the VKS exercise programme, including an online goal‐setting feature that provides personalised feedback, a PDF goal‐setting and recording sheet and a PDF exercise diary at a minimum.
5	To ensure that users with severe knee signs/symptoms and varying personal preferences and circumstances can safely engage with the VKS exercise programme.	Providing a flexible pre‐operative TKR exercise programme that is tailored to the needs of users with severe knee signs/symptoms and does not require nonhousehold equipment or facilities.
		Providing clear guidance about how to safely select, perform and progress exercises, including videos of relatable Patient Models demonstrating exercises at a minimum.
6	To ensure that users know how to make healthy lifestyle changes and build their motivation to do so.	Explaining the potential benefits of making healthy lifestyle changes, including for post‐operative recovery.
		Including brief guidance on making healthy lifestyle changes, with signposting to credible sources of further guidance.

Abbreviations: NHS, National Health Service; PDF, portable document format; TKR, total knee replacement; UK, United Kingdom; VGP, Virtual Knee School guiding principle; VKS, Virtual Knee School.

2. *Undertaking a behavioural analysis*.

Supporting Information: File 2 provides the behavioural analysis tables. The potential VKS features targeted all six COM‐B model components and employed six BCW intervention functions (education, persuasion, training, environmental restructuring, modelling and enablement). The BCW intervention functions not employed (incentivisation, coercion and restrictions) involve creating an expectation of external consequences or imposing external rules, which may reduce intrinsic motivation,^74^, ^75^ and thus were not considered appropriate for the VKS.

The potential VKS features employed 25 BCTs. Fourteen additional BCTs were identified from the systematic review by Safari et al.^42^ (Supporting Information: File 2). A comparison of the behavioural analysis tables with these 14 BCTs and the BCTTv1 did not lead to the inclusion of any extra BCTs. This was mainly because the behavioural analysis tables already included numerous BCTs identified through a rigorous process, so implementing these BCTs well was considered more of a priority than adding extra BCTs, which are likely to have been less contextually relevant.

No major inconsistencies between the behavioural analysis tables and VKS guiding principles were identified. However, the healthy lifestyle change behavioural analysis table was particularly extensive. Adding extra healthy lifestyle change‐related features to the VKS guiding principles to account for that was decided against to help ensure the VKS was not too complex/overwhelming for users.

3. *Developing a logic model*.

**Figure 2:**
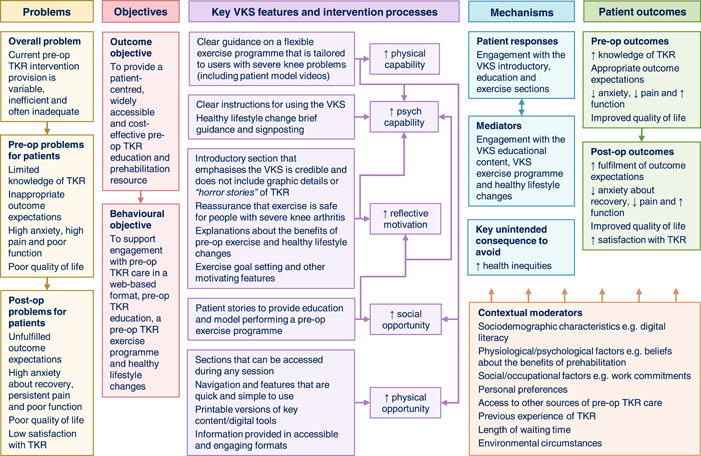
Virtual Knee School (VKS) logic model. Logic model of the VKS, including the problems it seeks to address, its main objectives, its key features and intervention processes, its proposed causal mechanisms, the intended patient outcomes and contextual moderators. To avoid overcrowding and ensure legibility, relationships between factors in different columns of the logic model are not shown. Postop, post‐operative; pre‐op, pre‐operative; psych, psychological; TKR, total knee replacement.

Figure 2 provides the VKS logic model. As this shows, the VKS aims to help address variations, inefficiencies, and inadequacies in current pre‐operative TKR intervention provision. The key VKS features target all the COM‐B model components except for automatic motivation. The intended patient responses to the VKS are proposed to dynamically interact with the VKS mediators. Some patients may be unable to access/effectively engage with websites; therefore, the key unintended consequence to avoid is increasing health inequities. The VKS mediators are proposed to improve numerous pre‐ and post‐operative patient outcomes. Various contextual moderators may affect patient outcomes both directly and indirectly by influencing VKS's proposed causal mechanisms.

### 3.2 VKS prototype development and refinement (Phase 4)

#### 3.2.1 VKS prototype summary

**Figure 3:**
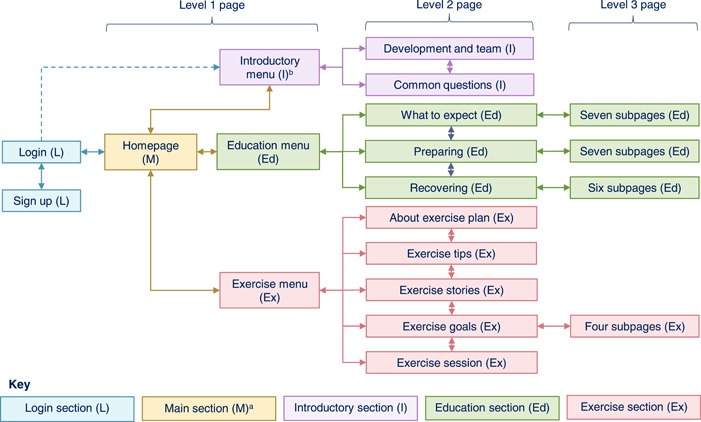
Virtual Knee School (VKS) prototype information architecture summary. Summary of the VKS prototype information architecture, showing the five website sections and three page levels. ^a^ The main section also included the following pages accessible via the header, footer or meganav (expandable menu): Help; Accessibility statement; Privacy and cookies policy; Other helpful websites; Contact us. ^b^ Users were tunnelled to the introductory section menu on their first login but not subsequent logins.

Figure 3 and Supporting Information: File 3 summarise the initial VKS prototype. A hybrid information architecture was employed. On their first login, users were tunnelled to the introductory section menu to help ensure they viewed a welcome video aimed at addressing key barriers to engagement with the VKS and its target behaviours. Users could then access the remaining sections in any order.

To account for users' varying preferences and needs, two tailoring strategies were employed.

1. *Computer‐tailoring*: this involves using computer algorithms to adapt an intervention's content/delivery to the individual user.^76^, ^77^ The key application of computer tailoring in the VKS prototype was in the goal‐setting feature, which provided personalised feedback based on the user's goal attainment.
2. *Self‐tailoring*: this involves offering choices so the user can adapt the intervention's content/delivery themselves.^78^ Multiple self‐tailoring strategies were employed in the VKS prototype. For example, the accessibility toolbar enabled users to change the language, text size and contrast; and the goal‐setting feature included the option to set a personal exercise goal.

#### 3.2.2 VKS prototype evaluation and refinement

The lead researcher approached 29 patients via the NHS teaching hospital and was contacted by six additional patients. Of these 35 patients, 24 were screened, 10 were invited to participate, and nine consented. Two participants withdrew after their first interview due to increased anxiety or serious health problems. Supporting Information: File 3 provides the participant flow chart and participants' characteristics. The relevant participant's pseudonym, age group, experience of TKR and confidence in using the Internet are provided for each illustrative quote.

##### Participants' overall views of the VKS prototype

Most participants were positive about the VKS prototype overall, making comments such as ‘I think it's an absolutely invaluable tool’. Key reported benefits included that it is comprehensive, realistic and reassuring; and would provide a constantly available resource to refer back to. One participant felt viewing the VKS before being listed for TKR would have facilitated her decision‐making and helped her identify questions to ask her consultant. Furthermore, three participants commented they would have liked to access the prototype pre‐ and post‐operatively:

And I would have loved, if I had been lying in bed afterwards, it would have been great to just be able to look up anything I thought about. (Arthur, 80–89, post‐TKR, very confident)

Feedback about the variety of exercises, accordions (expandable headings) and patient stories was particularly positive. Participants were also very complimentary about the ‘perfectly great videos’, valuing aspects such as their clarity and the option to add subtitles in other languages. Three participants specifically highlighted that the exercise videos were easier to follow than static images:

It's nice to have all the exercises videoed out, rather than just a diagram showing you where to move your hand next or where to move your leg next because I don't think they're very constructive a thing. Seeing videos like this is more beneficial. (Ella, 40–49, pre‐TKR, confident)

Participants generally felt the accessibility toolbar was useful, and a few emphasised they liked the ‘simple language’. Most participants also thought the website was clear and simple to use, even for people with lower digital literacy:

I liked the website, how it was organised. And it was very visual. Then if you're not very computer literate it's very practical. (Jessica, 50–59, pre‐TKR, neither confident nor unconfident)

In contrast, both participants who were unconfident in using the Internet felt the digital format did not meet their needs, as they found it anxiety‐provoking and/or too difficult to use:

[…] to me a website is alright if you can use these, but if you can't use them, it's just not helpful at all. (Vera, 70–79, post‐TKR, unconfident)

Both these and other participants emphasised the importance of providing support via alternative formats such as face‐to‐face care, a video or a booklet. Correspondingly, all four participants who viewed the portable document format (PDF) exercise booklet felt it was valuable:

I think that [exercise booklet]'s really good because I think, again, thinking about accessibility and people not having full‐time access to the Internet or laptop or whatever. (Naomi, 60–69, post‐TKR, very confident)

One participant who was confident in using the Internet also felt the VKS prototype did not meet his needs. This was mainly because he knew most of the information already and perceived the exercise programme as too easy. The latter appeared to be at least partly because he had ready access to a swimming pool, so was used to exercising in water. This participant also disliked the instructions on aspects such as how to use the website and play a video, which he found ‘a bit babyish’ and unnecessary:

[…] but it's just a bit, making me feel like, ooh, blooming heck, more load of rubbish, you know, I don't need all this. (Laurence, 60–69, pre‐TKR, confident)

Conversely, other participants provided positive feedback about the instructions. Conflicting feedback was also obtained about other content/features. For example, some participants felt the goal‐setting feature would support them to engage with the exercise programme. Reasons for this included that it would provide a focus and ‘something to kind of measure yourself against’. Many participants particularly liked the personalised feedback as they considered it encouraging, constructive and specific. In contrast, a few participants did not think they would use the goal‐setting feature. This appeared to be because they were already confident in their ability to adhere to their exercise programme. One participant also suggested that an individual's personality would influence whether they used the goal‐setting feature:

I think a lot of it's down to your personality, to be quite honest. I think there are people that would welcome it and think it's absolutely brilliant. There are other people that would think, well, I can't be bothered […] (Glen, 70–79, post‐TKR, confident)

Participants' opinions were also divided over the sign‐up/login process. Although many participants found the process easy, others found it difficult or required assistance to complete it. Additionally, some participants raised broader concerns about signing up, such as a fear of being sent lots of messages. Correspondingly, a few participants felt at least some of the VKS should be freely accessible without the need to sign up:

I think you should [make most of the VKS freely accessible], especially for… Most of the people will be older people who are not very computer literate and having to put passwords in, understanding lowercase and uppercase and with their stubbly arthritic fingers, like myself, they seem to go everywhere. (Haaniya, 60–69, pre‐ and post‐TKR, neither confident nor unconfident)

In contrast, other participants were quite happy with the idea of signing up or even preferred it, for example, due to feeling it would enable them to receive more personalised content.

##### Refinements to the VKS prototype

Multiple potential changes to the VKS prototype were identified, prioritised and implemented when appropriate (Table 6).

**Table 6:** Summary of the main changes made to the VKS prototype.

VKS prototype section/aspect	Issues^a^	Main changes
Design and overall content	Not realising it was possible to select the accordions (expandable headings).	The accordions' background colour was changed to blue to distinguish them from other website features. Text was added to highlight that users can select the accordions.
	Feeling there was too much text.	Restructuring some of the text into accordions.
	Having difficulty locating and/or using the accessibility toolbar.	The accessibility toolbar instructions were updated for clarity. The accessibility toolbar header was amended to display ‘Hide website accessibility tools’ when it was open and ‘Show website accessibility tools’ when it was closed.
	Being concerned about whether there was enough time to watch the videos.	The duration of each educational video was added to its title. (Durations were also added to the exercise video titles but were misunderstood as referring to the durations of the exercises themselves, so were subsequently removed).
	Believing the patient stories were from real‐life patients.	Text was added above the stories to explain that the stories were based on other patients' experiences.
Information architecture and navigation	Finding the tunnelling to the introductory section menu unhelpful/confusing.	The tunnelling was removed so that users went straight to the main homepage on their first login, and text was added to advise users to select the introductory section picture button if it was their first login.
	Feeling overwhelmed by the volume of content due to the education dropdown menu displaying the titles of all 24 education pages/subpages.	The education menu page was removed and the education subsections were promoted to full sections, limiting the number of page titles displayed at once to a maximum of eight.
	Not realising it was possible to select the small triangles to display lower level pages when using the meganav on a mobile device in portrait orientation.	The size of the triangles in the meganav was increased.
	Feeling confused by the back and next buttons both going to the same page if the user accessed the last page in a section from the section menu.	The next buttons were removed from the final page in each section.
	Feeling extra hyperlinks would be useful for quickly checking other pages, and feeling confused about whether words in bold were hyperlinks.	Extra hyperlinks were added where appropriate.
Login section	Mistyping characters leading to the two passwords entered on the sign‐up page not matching or the password entered on the login page being incorrect.	‘Show password’ options were added to the sign‐up and login pages.
Main section	Feeling the main homepage did not make it clear that the website had three main sections.	The location and formatting of the button to the introductory section menu was amended so that the homepage included three picture buttons, corresponding with the three website sections. Text was added to explain how many sections the website has.
	Feeling it should be clearer that the website provides information related to the peri‐ and post‐operative phases, rather than just the pre‐operative phase.	The banner text on the main homepage and the text on the login page were updated to explain that the website is designed to help patients ‘prepare for before, during and after’ TKR surgery.
	Feeling the three homepage picture buttons did not indicate where to find the information the user wanted.	The three education subsections were promoted to full sections so that the homepage included five picture buttons, corresponding with the five website sections, hence providing a greater level of detail about the information available. The title of the expectations section was changed from ‘What to expect’ to ‘About your operation’ for clarity.
	Feeling a link to the ‘Contact us’ page should be included in the website footer for consistency with other websites.	A link to the ‘Contact us’ page was added to the website footer.
	Considering using the VKS email address to ask questions about the user's own operation.	Text was added to clarify that users should contact their own care team for questions about their own operation and the VKS email address is only for questions about the VKS itself.
Introductory section	Feeling there was too much information on the introductory section menu.	The instructions on how to use the accessibility toolbar and website were moved into accordions.
	Feeling confused by the instructions on how to use the website.	Separate instructions were provided about how to use the website on computers and mobile devices. Labelled screenshots were added to the instructions.
	Finding the PDF of the Phase 1b modified Delphi study recommendations too detailed and ‘very confusing’.	The document was deleted from the ‘VKS development and team’ page and a link to the Phase 1b journal publication was added to the ‘Other helpful websites’ page instead.
	Feeling it would be helpful to amend the wording of certain answers on the ‘Common questions’ page.	Minor text amendments were made to specific answers, for example, to highlight that exercising can help to relieve knee stiffness.
	Feeling it would be helpful to cover what to do if the user has bilateral knee problems on the ‘Common questions’ page.	An accordion was added to explain that the VKS exercise programme is appropriate for people with bilateral knee problems.
Education section	Requesting further information about specific topics.	Hyperlinks to other pages of the prototype were added where appropriate, for example, a hyperlink to the ‘Recovering from your operation’ menu was added to the ‘After your hospital stay’ page. Minor text amendments were made where appropriate, for example, text was added to the ‘Planning your return to work’ page to explain why users may want to keep their original fit note.
	Feeling the ‘Goal setting’ page should provide more encouragement for users who do not meet their goals.	Text was added to provide more encouragement for users who do not meet their goals.
	Wanting post‐operative goals to look forward to and ‘something visual’.	An accordion with examples of post‐operative goals to look forward to and a photograph of a beach was added.
Exercise section	Feeling confused about whether the exercise section was for the pre‐ or post‐operative phase.	‘Pre‐op’ was added to the exercise section title. The introductory text on the exercise section menu was amended for clarity.
	Highlighting queries or concerns about specific aspects of the exercise section text.	Minor text amendments were made where appropriate, for example, text was added to the exercise instructions to advise users to build up to exercising every day if they feel able to.
	Thinking the exercise category titles related to the videos above them rather than below them.	A horizontal line was added above and below each exercise category. The exercises were labelled to correspond with their category, for example, ‘Category 1’ exercises were labelled as ‘1a Seated Marching’, ‘1b Walking on the spot’ and so on.
	Missing the ‘Submit’ button on the goal‐setting and review forms.	Text was added to the goal‐setting and review forms to explain that users need to select the ‘Submit’ button before proceeding to the next page.
	Entering numbers in the goal‐setting form as words rather than numerals.	Text was added to the goal‐setting form to advise users to enter numbers as numerals rather than words.
	Finding it challenging to set appropriate exercise goals due to unfamiliarity with the VKS exercise programme.	The exercise pages were reordered so that the ‘Carry out an exercise session’ page was before the goal‐setting pages. Text was added to the ‘Set your exercise goals’ page to advise users to try carrying out a VKS exercise session before setting their goals.

Abbreviations: PDF, portable document format; VKS, Virtual Knee School.

^a^ Supporting Information: File 3 provides an example quote for each issue.

Two of the most substantial changes involved amending the VKS prototype's information architecture. First, the tunnelling to the introductory section menu was removed because two of the four participants who trialled it found it unhelpful/confusing. One participant related this to the relatively large volume of text on the introductory menu, which she felt could be ‘off‐putting’. The other participant felt all websites should open at the homepage because ‘that's the starting point’. The second major information architecture change was primarily made in response to comments about the education dropdown menu. This displayed the titles of all 24 education pages/subpages, making the volume of content seem overwhelming:

When you see all these sections, you think it's going to be a mammoth, but I like the fact that it's short, it's straight to the point. (Ella, 40–49, pre‐TKR, confident)

This was addressed by removing the education menu page and promoting the education subsections to full sections. Each education section then had a separate dropdown menu, limiting the number of page titles displayed to a maximum of eight.

Most other changes were more minor adaptions to the design or content. For example, ‘pre‐op’ was added to the exercise section title to help avoid confusion about the exercise programme timing. Subsequent feedback suggested this change was successful:

[…] because you've put pre‐op exercise plan, it is made clear it's pre‐op, not post‐op. (Jessica, 50–59, pre‐TKR, neither confident nor unconfident)

##### Examples of issues that were not fully resolved

Whilst most changes appeared to be successful, some issues were not fully resolved. For example, after amending the accessibility toolbar instructions and header for clarity, one participant still felt the accessibility toolbar was too complex for her:

Well, it is good for people who are very literate, fluent in computer and anything it's alright, but I'm at the creeping stage. […] I'm still bottle fed. (Zuri, 70–79, pre‐ and post‐TKR, unconfident)

A few issues were not fully resolved because participants missed extra text that had been added. There were also some issues that were not addressed to avoid contradicting other priorities/feedback. For example, one participant felt patients may not have time/be able to make lifestyle changes pre‐operatively, so was concerned that the healthy lifestyle information may risk ‘setting you up to fail’ Removing the healthy lifestyle information to address this would have been inconsistent with VKS guiding principle six. Furthermore, other participants felt the healthy lifestyle guidance was valuable:

It's all good general stuff that relates specifically to the operation but has much wider implications. (Glen, 70–79, post‐TKR, confident)

### 3.3 Meta‐inferences

**Figure 4:**
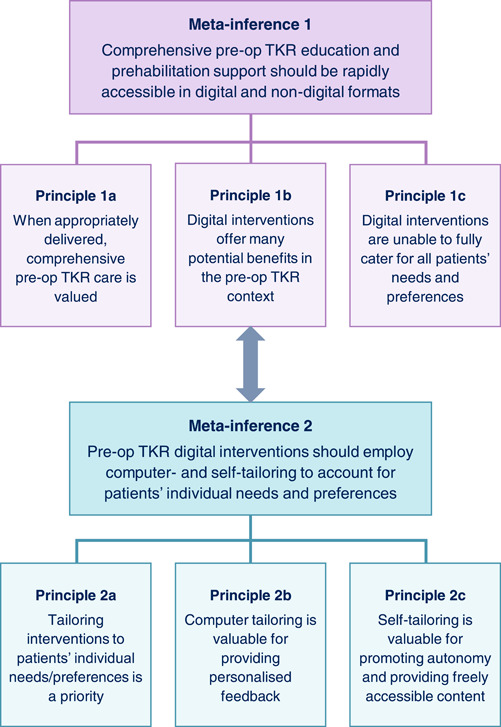
Meta‐inferences schematic diagram. Summary of the meta‐inferences generated by integrating the findings of all the project phases. The three principles underpinning each meta‐inference, and the intersection between the two meta‐inferences, are included. Pre‐op, pre‐operative; TKR, total knee replacement.

Two intersecting meta‐inferences were generated, each of which is underpinned by three principles and provides a recommendation for clinical practice and future research (Figure 4). A brief overview of the rationale for each meta‐inference is provided below.

#### 3.3.1 Meta‐inference 1: Comprehensive pre‐operative TKR education and prehabilitation support should be rapidly accessible in digital and non‐digital formats

This project's findings suggest patients and health professionals generally perceive comprehensive pre‐operative TKR support as valuable, but there is a risk of overwhelming patients with too much information. Delivering information appropriately appears key to addressing this risk. For example, Phase 4 participants felt the accordions were useful for reducing the volume of text displayed on the VKS prototype. Another important finding was that digital interventions offer multiple potential benefits in the pre‐operative TKR context. A range of potential benefits were identified across all the project phases. These included increasing service efficiency, providing tailored support, allowing rapid information provision, and providing a constantly available resource to refer back to. Benefits of specific digital features were also identified, such as exercise videos being easier to follow than static images. Conversely, it was evident throughout this project that digital interventions are unable to fully cater for all patients' needs and preferences. For example, some patients find digital interventions difficult to use and anxiety‐provoking, or simply prefer paper‐based alternatives. Providing support in both digital and non‐digital formats is therefore recommended.

#### 3.3.2 Meta‐inference 2: Pre‐operative TKR digital interventions should employ computer‐ and self‐tailoring to account for patients' individual needs and preferences

Meta‐inference 2 focuses on digital intervention tailoring. It, therefore, intersects with meta‐inference 1, which highlights a potential benefit of digital interventions is that they can provide tailored support. The importance of tailoring pre‐operative TKR interventions to patients' individual needs and preferences was emphasised throughout this project. For example, the VKS guiding principles state the VKS education and exercise programme should account for users' varying preferences and needs/circumstances. To help address this, computer‐ and self‐tailoring strategies were employed in the VKS prototype. Some Phase 4 participants particularly liked the VKS goal‐setting feature, supporting the use of computer‐tailoring for providing personalised feedback. Phase 4 participants also provided positive comments about features such as the accessibility toolbar, highlighting the value of self‐tailoring strategies. Another key benefit of self‐tailoring strategies is that they do not rely on users logging in, unlike many computer‐tailoring strategies.

## 4 DISCUSSION

This paper reports how a VKS digital intervention for patients awaiting TKR was systematically developed using an evidence‐, theory‐ and person‐based approach. The findings of three intervention planning phases were combined with numerous PPI activities to create a VKS prototype. Evaluating how patients used the prototype and exploring their perspectives of it enabled key usability problems and broader concerns about the prototype to be identified. Most of these were successfully addressed. Many participants considered the VKS a valuable resource, but a minority felt its digital format or content did not meet their individual needs. Integrating the findings of all the project phases generated two meta‐inferences, each of which provides a recommendation on pre‐operative TKR care for clinical practice and future research.

The diversity of feedback obtained about the acceptability of the VKS prototype is a key finding of this project. Acceptability is a broad concept, encompassing components such as perceived effectiveness, usability and burden.^79^ Most participants appeared to find the VKS prototype acceptable overall because they valued its content and considered it relatively easy to use. Conversely, three participants felt the prototype's acceptability was low in their specific context. For one participant, this appeared to relate mainly to the prototype's perceived effectiveness, as he thought its content was too basic. Arguably, this does not present a major concern for the potential value of the VKS as this individual had already obtained and acted on relevant health information, suggesting he had high health literacy. Ensuring the VKS is appropriate for individuals with low health literacy is more of a priority because low musculoskeletal health literacy is associated with worse outcomes post‐TKR,^7^ and digital interventions have the potential to improve health literacy.^23^

The other two participants who perceived the VKS as less acceptable related this to the digital delivery format, which they found anxiety‐provoking and/or too difficult to use. This demonstrates that digital interventions are unlikely to meet all patients' needs, even when their development involves extensive patient input and prioritises accessibility/inclusion. This is a major concern for health equity, as patients at risk of digital exclusion often have the greatest health needs.^22^ One option to address this would be to employ digital inclusion strategies, such as signposting patients who are given a digital health intervention to third‐sector digital skills training programmes. This may be particularly valuable because gaining digital skills is likely to have positive effects on other areas of patients' lives.^22^ As participants in this project highlighted, it is also essential to offer non‐digital formats to account for patients who remain unable/unwilling to use digital interventions.

Usability incorporates the effectiveness, efficiency and satisfaction with which users can achieve their objectives when using an intervention.^80^ Many of the VKS prototype's initial usability problems were linked to efficiency and satisfaction. For example, a couple of participants found the tunnelling to the introductory section unhelpful/confusing, but it did not prevent them from navigating the prototype. There were also instances where participants were unable to achieve their objectives effectively. Most notably, a few participants could not complete the sign‐up/login process independently. Some participants also raised broader concerns about signing up, such as a fear of being sent lots of messages. Similar issues have been highlighted in previous research^81^ and could be addressed by making some of the intervention content freely accessible without the need to sign up. This approach could feasibly be implemented in clinical practice. However, it could pose problems from an evaluation perspective. For example, control group participants could potentially access the freely accessible content, increasing the risk of contamination bias.^82^

This project builds on previous studies demonstrating the value of using an evidence‐, theory‐ and person‐based approach to develop digital interventions.^39^, ^40^, ^41^ There are some notable similarities between this project's findings and those of previous studies. For example, when refining their digital intervention for cancer survivors, Bradbury et al.^41^ made the names of buttons in the intervention sections more descriptive to help avoid confusion. Similarly, the VKS exercise section title was amended to include ‘pre‐op’. This emphasises the importance of ensuring that digital intervention content is self‐evident or at least self‐explanatory.^83^ This project expands on previous evidence‐, theory‐ and person‐based approach intervention development studies by demonstrating how aspects of the approach can be adapted. For example, this project involved developing bespoke criteria for prioritising potential features and prototype changes (Tables 2 and 4).

### 4.1 Strengths and limitations

The systematic and transparent approach used to develop the VKS is a key strength of this project. Furthermore, generating meta‐inferences provided greater insights than would have been gained by considering each phase in isolation. The central role of PPI was another strength. By being involved in multiple activities, the PAG PPI members developed a thorough understanding of the project and provided highly valuable input. This complemented the qualitative research, which involved patients who were unfamiliar with the project and hence offered ‘fresh’ perspectives.^84^

Only including three PPI members in the PAG limited the group's diversity. Similarly, there were some limitations with the diversity of the think‐aloud interview sample. Diversity was obtained in key characteristics, such as age, confidence in using the Internet and educational level (Supporting Information: File 3). Some ethnic diversity was obtained, but only patients able to communicate in English were eligible. Furthermore, few participants had a disability or health condition that could affect their ability to use a website or carry out gentle exercises, so it was not possible to comprehensively explore the accessibility of the VKS. Additional limitations of this project were that all the phases relied on some subjective judgements (e.g., during the data analysis), and participants did not have the opportunity to try using the VKS prototype independently or implementing the intended health behaviour changes.

### 4.2 Implications for practice and future research

Both meta‐inferences generated in this project provide a recommendation for clinical practice and future research. The first recommendation states comprehensive pre‐operative TKR education and prehabilitation support should be rapidly accessible in digital and non‐digital formats. The project's findings highlight strategies for addressing this, such as ensuring that all sections of digital interventions are rapidly accessible and providing pre‐operative TKR support via a booklet. Future research focused on identifying how to optimise the implementation of pre‐operative TKR care in digital and non‐digital formats would be valuable. As discussed above, this could include incorporating digital inclusion strategies.

The second recommendation suggests pre‐operative TKR digital interventions should employ computer‐ and self‐tailoring to account for patients' individual needs and preferences. Complementary benefits of these tailoring strategies were identified and suggest it would be helpful to employ the following:

1. *Self‐tailoring strategies in isolation to deliver freely accessible content*: This could include offering features such as an accessibility toolbar (for changing the language, text size and contrast) and accordions (for providing optional extra text); providing a flexible exercise programme with a choice of different exercises; and delivering content using more than one format (e.g., exercise videos and a PDF exercise booklet).
2. *Computer‐tailoring strategies combined with self‐tailoring strategies, where appropriate, deliver features that provide personalised feedback*: This could include providing a goal‐setting feature that offers a choice of goals and provides personalised feedback based on the user's goal attainment. It could also include providing healthy lifestyle screening features, such as an alcohol consumption screening feature that provides personalised feedback about whether the user is meeting low‐risk drinking guidelines. This study's findings highlight the importance of ensuring that any feedback provided is encouraging, constructive and specific.

Future research of pre‐operative TKR digital interventions could explore other computer‐tailoring strategies, such as tailoring the message frame to patients' information processing styles.^77^

Overall, this project's findings suggest the VKS is a potentially valuable resource and warrants further research. Conducting a randomised feasibility study to determine if/how to progress to a randomised controlled trial (RCT) would be a logical next step. Pursuing this option would be a lengthy process. This presents a tension with PAG members' feedback, which suggested the priority should be to rapidly implement the VKS. In light of this feedback and the limitations of RCTs, considering alternative evaluation options is warranted. For example, conducting a realist evaluation could be valuable for exploring how the VKS works/does not work for specific groups of patients in specific contexts.^85^ Given the concerns about digital exclusion highlighted above, and the intersection of digital exclusion with other social determinants of health,^22^ it is a priority to ensure that any future research into the VKS explores its impact on health inequities.

## 5 CONCLUSIONS

This project systematically integrated evidence, theory, and stakeholders' perspectives to develop a novel pre‐operative TKR education and prehabilitation digital intervention, the ‘Virtual Knee School’. The central role of PPI throughout the project helped to optimise the acceptability and inclusivity of the research procedures and VKS prototype. Feedback from diverse participants enabled the prototype to be iteratively refined. The findings suggest the VKS is a promising resource, but its digital format is unlikely to meet all patients' individual needs. Future research of the VKS is therefore warranted and should include exploring its impact on health inequities. Integrating the findings of all the project phases emphasised the importance of providing comprehensive, rapidly accessible pre‐operative TKR support in digital and non‐digital formats; and suggested that pre‐operative TKR digital interventions should employ computer‐ and self‐tailoring to account for patients' individual needs and preferences.

## AUTHOR CONTRIBUTIONS

Anna M. Anderson led the study conception, study design, data acquisition, data analysis, data interpretation, drafting the manuscript and revising the manuscript. Gretl A. McHugh, Christine Comer and Anthony C. Redmond contributed to the study conception, study design, data interpretation and revision of the manuscript. Judith Joseph, Toby O Smith and Lucy Yardley contributed to the study design, data interpretation and revising of the manuscript. All authors read and approved the final manuscript.

## CONFLICT OF INTEREST STATEMENT

The authors declare no conflict of interest.

## Supporting information

Supporting information.

Supporting information.

Supporting information.

## Data Availability

Data that support the findings of this study are available in the Supporting Information: Material of this article, where appropriate. Additional data supporting the findings of this study are available on request from the corresponding author but are not publicly available due to privacy or ethical restrictions.

## References

[1] Price AJ , Alvand A , Troelsen A , et al. Knee replacement. Lancet. 2018;392(10158):1672‐1682. 30496082 10.1016/S0140-6736(18)32344-4

[2] NJR Editorial Board and Contributors. National Joint Registry 18th Annual Report 2021: Surgical data to 31 December 2020. National Joint Registry; 2021. https://reports.njrcentre.org.uk/Portals/0/PDFdownloads/NJR%2018th%20Annual%20Report%202021.pdf

[3] Beswick AD , Wylde V , Gooberman‐Hill R , Blom A , Dieppe P . What proportion of patients report long‐term pain after total hip or knee replacement for osteoarthritis? A systematic review of prospective studies in unselected patients. BMJ Open. 2012;2(1):e000435e. 10.1136/bmjopen-2011-000435 PMC3289991 22357571

[4] DeFrance MJ , Scuderi GR . Are 20% of patients actually dissatisfied following total knee arthroplasty? A systematic review of the literature. J Arthroplasty. 2023;38(3):594‐599. 36252743 10.1016/j.arth.2022.10.011

[5] Kahlenberg CA , Nwachukwu BU , McLawhorn AS , Cross MB , Cornell CN , Padgett DE . Patient satisfaction after total knee replacement: a systematic review. HSS J. 2018;14(2):192‐201. 29983663 10.1007/s11420-018-9614-8 PMC6031540

[6] Lewis GN , Rice DA , McNair PJ , Kluger M . Predictors of persistent pain after total knee arthroplasty: a systematic review and meta‐analysis. Br J Anaesth. 2015;114(4):551‐561. 25542191 10.1093/bja/aeu441

[7] Narayanan AS , Stoll KE , Pratson LF , Lin F‐C , Olcott CW , Del Gaizo DJ . Musculoskeletal health literacy is associated with outcome and satisfaction of total knee arthroplasty. J Arthroplasty. 2021;36(7S):S192‐S197. 33812715 10.1016/j.arth.2021.02.075

[8] Hafkamp FJ , Gosens T , de Vries J , den Oudsten BL . Do dissatisfied patients have unrealistic expectations? A systematic review and best‐evidence synthesis in knee and hip arthroplasty patients. EFORT Open Rev. 2020;5(4):226‐240. 32377391 10.1302/2058-5241.5.190015 PMC7202041

[9] Judge A , Carr A , Price A , et al. The Impact of the Enhanced Recovery Pathway and Other Factors on Outcomes and Costs Following Hip and Knee Replacement: Routine Data Study. NIHR Journals Library; 2020. 32040280

[10] Tolk JJ , Janssen RPA , M. HT , van der Steen MC , Bierma‐Zeinstra SMA , Reijman M. The influence of expectation modification in knee arthroplasty on satisfaction of patients: a randomized controlled trial. Bone Joint J. 2021;103‐B(4):619‐626. 10.1302/0301-620X.103B4.BJJ-2020-0629.R3 33789470

[11] Punnoose A , Claydon‐Mueller LS , Weiss O , Zhang J , Rushton A , Khanduja V . Prehabilitation for patients undergoing orthopedic surgery: a systematic review and meta‐analysis. JAMA Network Open. 2023;6(4):e238050‐e. 37052919 10.1001/jamanetworkopen.2023.8050 PMC10102876

[12] De Klerk TC , Dounavi DM , Hamilton DF , Clement ND , Kaliarntas KT . Effects of home‐based prehabilitation on pre‐ and postoperative outcomes following total hip and knee arthroplasty. Bone & Joint Open. 2023;4(5):315‐328. 37142259 10.1302/2633-1462.45.BJO-2023-0021 PMC10159731

[13] Wu Z , Wang Y , Li C , et al. Preoperative strength training for clinical outcomes before and after total knee arthroplasty: a systematic review and meta‐analysis. Front Surg. 2022;9:879593. 35937597 10.3389/fsurg.2022.879593 PMC9349363

[14] National Institute for Health and Care Excellence. Joint Replacement (Primary): Hip, Knee and Shoulder (NICE Guideline [NG157]). NICE; 2020. 32881469

[15] Getting It Right in Orthopaedics: reflecting on success and reinforcing improvement. Getting It Right First Time. February 2020. Accessed July 7, 2020. https://gettingitrightfirsttime.co.uk/wp-content/uploads/2020/02/GIRFT-orthopaedics-follow-up-report-February-2020.pdf

[16] Scott NB , McDonald D , Campbell J , et al. The use of enhanced recovery after surgery (ERAS) principles in Scottish orthopaedic units—an implementation and follow‐up at 1 year, 2010–2011: a report from the Musculoskeletal Audit, Scotland. Arch Orthop Trauma Surg. 2013;133(1):117‐124. 23070220 10.1007/s00402-012-1619-z

[17] Grosso MJ , Courtney PM , Kerr JM , Della Valle CJ , Huddleston JI . Surgeons' preoperative work burden has increased before total joint arthroplasty: a survey of AAHKS members. J Arthroplasty. 2020;35(6):1453‐1457. 32057605 10.1016/j.arth.2020.01.079

[18] NHS England and NHS Improvement . Delivery Plan for Tackling the COVID‐19 Backlog of Elective Care. PAR C1466. NHS; 2022. Accessed May 29, 2023. https://www.england.nhs.uk/coronavirus/wp-content/uploads/sites/52/2022/02/C1466-delivery-plan-for-tackling-the-covid-19-backlog-of-elective-care.pdf

[19] National Health Service . The NHS Long Term Plan, Version 1.2. NHS; 2019. Accessed February 4, 2022. https://www.longtermplan.nhs.uk/publication/nhs-long-term-plan/

[20] Shah N , Costello K , Mehta A , Kumar D . Applications of digital health technologies in knee osteoarthritis: narrative review. JMIR Rehabil Assist Technol. 2022;9(2):e33489. 35675102 10.2196/33489 PMC9218886

[21] Michie S , Yardley L , West R , Patrick K , Greaves F . Developing and evaluating digital interventions to promote behavior change in health and health care: recommendations resulting from an international workshop. J Med Internet Res. 2017;19(6):e232. 28663162 10.2196/jmir.7126 PMC5509948

[22] Davies AR , Honeyman M , Gann B . Addressing the digital inverse care law in the time of COVID‐19: potential for digital technology to exacerbate or mitigate health inequalities. J Med Internet Res. 2021;23(4):e21726. 33735096 10.2196/21726 PMC8030655

[23] Davaris MT , Bunzli S , Trieu J , Dowsey MM , Choong PF . The role of digital health interventions to improve health literacy in surgical patients: a narrative review in arthroplasty. ANZ J Surg. 2022;92(10):2474‐2486. 35924880 10.1111/ans.17931

[24] Bahadori S , Wainwright TW , Ahmed OH . Smartphone apps for total hip replacement and total knee replacement surgery patients: a systematic review. Disabil Rehabil. 2020;42(7):983‐988. 30362363 10.1080/09638288.2018.1514661

[25] Ng MK , Emara AK , Molloy RM , Krebs VE , Mont M , Piuzzi NS . YouTube as a source of patient information for total knee/hip arthroplasty: quantitative analysis of video reliability, quality, and content. J Am Acad Orthop Surg. 2020;29(20):e1034‐e1044. 10.5435/JAAOS-D-20-00910 33252551

[26] Karimi AH , Shah A , Hecht 2nd CJ , Burkhart RJ , Acuña AJ , Kamath AF . Readability of online patient education materials for total joint arthroplasty: a systematic review. J Arthroplasty. 2023;5403(23):00052‐00059. 10.1016/j.arth.2023.01.032 36716898

[27] Anderson AM . *Development of a pre‐operative education and prehabilitation digital intervention for patients awaiting total knee replacement: a Virtual Knee School*. PhD thesis. University of Leeds; 2022.

[28] Duncan E , O'Cathain A , Rousseau N , et al. Guidance for reporting intervention development studies in health research (GUIDED): an evidence‐based consensus study. BMJ Open. 2020;10(4):e033516. 10.1136/bmjopen-2019-033516 PMC7245409 32273313

[29] Anderson AM , Drew BT , Antcliff D , et al. Content and delivery of pre‐operative interventions for patients undergoing total knee replacement: a rapid review. Syst Rev. 2022;11(1):184. 36050795 10.1186/s13643-022-02019-x PMC9436722

[30] Anderson AM , Comer C , Smith TO , et al. Consensus on pre‐operative total knee replacement education and prehabilitation recommendations: a UK‐based modified Delphi study. BMC Musculoskelet Disord. 2021;22(1):352. 33853564 10.1186/s12891-021-04160-5 PMC8044503

[31] Craig P , Dieppe P , Macintyre S , Michie S , Nazareth I , Petticrew M . Developing and Evaluating Complex Interventions. Medical Research Council; 2006.

[32] Yardley L , Morrison L , Bradbury K , Muller I . The person‐based approach to intervention development: application to digital health‐related behavior change interventions. J Med Internet Res. 2015;17(1):e30. 25639757 10.2196/jmir.4055 PMC4327440

[33] Yardley L , Ainsworth B , Arden‐Close E , Muller I . The person‐based approach to enhancing the acceptability and feasibility of interventions. Pilot Feasibility Stud. 2015;1:37. 27965815 10.1186/s40814-015-0033-z PMC5153673

[34] Creswell JW , Plano Clark VL . Designing and Conducting Mixed Methods Research. 3rd ed. SAGE Publications; 2018.

[35] Fetters MD , Curry LA , Creswell JW . Achieving integration in mixed methods designs‐principles and practices. Health Serv Res. 2013;48(6) (part 2):2134‐2156. 24279835 10.1111/1475-6773.12117 PMC4097839

[36] Tashakkori A , Teddlie C . Integrating qualitative and quantitative approaches to research. In: Bickman L , Rob DJ eds. The SAGE Handbook of Applied Social Research Methods. SAGE Publications; 2009:283‐317. 10.4135/9781483348858.n9

[37] Michie S , van Stralen MM , West R . The behaviour change wheel: a new method for characterising and designing behaviour change interventions. Implement Sci. 2011;6(1):42. 21513547 10.1186/1748-5908-6-42 PMC3096582

[38] Michie S , Richardson M , Johnston M , et al. The Behavior Change Technique Taxonomy (v1) of 93 hierarchically clustered techniques: building an international consensus for the reporting of behavior change interventions. Ann Behav Med. 2013;46(1):81‐95. 23512568 10.1007/s12160-013-9486-6

[39] Band R , Bradbury K , Morton K , et al. Intervention planning for a digital intervention for self‐management of hypertension: a theory‐, evidence‐ and person‐based approach. Implement Sci. 2017;12(1):25. 28231840 10.1186/s13012-017-0553-4 PMC5324312

[40] Greenwell K , Sivyer K , Vedhara K , et al. Intervention planning for the REDUCE maintenance intervention: a digital intervention to reduce reulceration risk among patients with a history of diabetic foot ulcers. BMJ Open. 2018;8(5):e019865. 10.1136/bmjopen-2017-019865 PMC5961606 29779008

[41] Bradbury K , Steele M , Corbett T , et al. Developing a digital intervention for cancer survivors: an evidence‐, theory‐ and person‐based approach. NPJ Digit Med. 2019;2:85. 31508496 10.1038/s41746-019-0163-4 PMC6718425

[42] Safari R , Jackson J , Sheffield D . Digital self‐management interventions for people with osteoarthritis: systematic review with meta‐analysis. J Med Internet Res. 2020;22(7):e15365. 32706657 10.2196/15365 PMC7428148

[43] Rehfuess EA , Booth A , Brereton L , et al. Towards a taxonomy of logic models in systematic reviews and health technology assessments: a priori, staged, and iterative approaches. Res Synth Methods. 2018;9(1):13‐24. 28677339 10.1002/jrsm.1254

[44] Moore G , Audrey S , Barker M , et al. Process Evaluation of Complex Interventions: UK Medical Research Council (MRC) Guidance. MRC; 2014. 10.1136/bmj.h1258 PMC4366184 25791983

[45] Mills T , Lawton R , Sheard L . Advancing complexity science in healthcare research: the logic of logic models. BMC Med Res Methodol. 2019;19(1):55. 30871474 10.1186/s12874-019-0701-4 PMC6419426

[46] Person‐Based Approach . The Person‐Based Approach to Intervention Planning. PBA; 2022. Accessed January 6, 2022. https://www.personbasedapproach.org/intervention_planning.html

[47] Bradbury K , Watts S , Arden‐Close E , Yardley L , Lewith G . Developing digital interventions: a methodological guide. Evid Based Complement Alternat Med. 2014;2014:1‐7. 10.1155/2014/561320 PMC3932254 24648848

[48] Kuhn J. Decrypting the MoSCoW analysis. Vol. 5.44. *DITY Weekly Newsletter*. 2009. http://www.itsmsolutions.com/newsletters/DITYvol5iss44.pdf

[49] The British Standards Institution . PAS 277: Health and Wellness Apps—Quality Criteria Across the Life Cycle—Code of Practice. BSI Standards Ltd; 2015.

[50] World Wide Web Consortium . Web Content Accessibility Guidelines (WCAG) 2.1. W3C; 2018. Accessed September 7, 2019. https://www.w3.org/TR/WCAG21/

[51] University of Leeds . Website Regulations. University of Leeds; 2022. Accessed January 12, 2022. https://comms.leeds.ac.uk/websites/website-regulations/

[52] National Institute for Health and Care Excellence . Osteoarthritis: Care and Management (Clinical Guideline [CG177]). NICE; 2014. 31869054

[53] Department of Health and Social Care, Welsh Government, Department of Health, Scottish Government . UK Chief Medical Officers' Low Risk Drinking Guidelines. Department of Health and Social Care, Welsh Government, Department of Health, Scottish Government; 2016. Accessed January 3, 2021. https://assets.publishing.service.gov.uk/government/uploads/system/uploads/attachment_data/file/545937/UK_CMOs__report.pdf

[54] Department of Health and Social Care, Welsh Government, Department of Health, Scottish Government . UK Chief Medical Officers' Physical Activity Guidelines. Department of Health and Social Care, Welsh Government, Department of Health, Scottish Government; 2019. Accessed December 5, 2019. https://www.gov.uk/government/publications/physical-activity-guidelines-uk-chief-medical-officers-report

[55] American College of Sports Medicine . ACSM's Guidelines for Exercise Testing and Prescription. 10th ed. Lippincott Williams and Wilkins; 2017.

[56] Welcome to GOV.UK. GOV.UK; 2023. Accessed July 29, 2023. https://www.gov.uk/

[57] National Health Service . We're Here For You. NHS; 2023. Accessed July 29, 2023. https://www.nhs.uk/

[58] National Joint Registry . Celebrating 20 Years of the Work of the Registry in Improving Outcomes for Patients. NJR; 2023. Accessed July 29 2023. https://www.njrcentre.org.uk/

[59] Versus Arthritis . We Are Here For You. Versus Arthritis; 2023. Accessed July 29, 2023. https://www.versusarthritis.org/

[60] Royal College of Anaesthetists . Welcome to the Royal College of Anaesthetists. Royal College of Anaesthetists; 2023. Accessed July 29, 2023. https://www.rcoa.ac.uk/

[61] The Royal College of Surgeons of England . Together, We're Changing the Face of Surgery. The Royal College of Surgeons of England; 2023. Accessed August 9, 2023. https://www.rcseng.ac.uk/

[62] South Tees Hospitals NHS Foundation Trust, York Trials Unit, University of Nottingham . OPAL Return to Work Program: Support for Returning to Work. South Tees Hospitals NHS Foundation Trust, York Trials Unit, University of Nottingham; 2021. Accessed January 18, 2023. https://sites.google.com/view/opalreturntowork/support-for-returning-to-work

[63] Frank . We listen. We guide. We collaborate . Frank; 2021. Accessed July 1, 2021. https://frankltd.co.uk/

[64] O'Cathain A , Croot L , Duncan E , et al. Guidance on how to develop complex interventions to improve health and healthcare. BMJ Open. 2019;9(8):e029954. 10.1136/bmjopen-2019-029954 PMC6701588 31420394

[65] Pearson J , Walsh N , Carter D , Koskela S , Hurley M . Developing a web‐based version of an exercise‐based rehabilitation program for people with chronic knee and hip pain: a mixed methods study. JMIR Res Protoc. 2016;5(2):e67. 27197702 10.2196/resprot.5446 PMC4891573

[66] Bradbury K , Morton K , Band R , et al. Using the person‐based approach to optimise a digital intervention for the management of hypertension. PLoS One. 2018;13(5):e0196868. 29723262 10.1371/journal.pone.0196868 PMC5933761

[67] van den Haak MJ , de Jong MDT , Schellens PJ . Evaluation of an informational web site: three variants of the think‐aloud method compared. Tech Commun. 2007;54(1):58‐71.

[68] Alhadreti O , Mayhew PJ . To intervene or not to intervene: an investigation of three think‐aloud protocols in usability testing. J Usability Stud. 2017;12(3):111‐132.

[69] Zhao T , McDonald S. Keep talking: an analysis of participant utterances gathered using two concurrent think‐aloud methods. In: *Proceedings of the 6th Nordic Conference on Human‐Computer Interaction, 16‐20 October 2010. Reykjavik, Iceland*. ACM; 2010:581‐590. 10.1145/1868914.1868979

[70] Tamler H . How (much) to intervene in a usability testing session. Common Ground. 1998;8(3):11‐15.

[71] Person‐Based Approach . Useful Resources. PBA; 2019. Accessed September 7, 2019. https://www.personbasedapproach.org/resources.html

[72] Person‐Based Approach . Key Steps to Making and Using a Table of Changes. PBA. Accessed May 6, 2022. https://www.personbasedapproach.org/table_of_changes.html

[73] Essery R , Pollet S , Smith KA , et al. Planning and optimising a digital intervention to protect older adults' cognitive health. Pilot Feasibility Stud. 2021;7(1):158. 34407886 10.1186/s40814-021-00884-2 PMC8371874

[74] Ryan RM , Deci EL . Self‐determination theory and the facilitation of intrinsic motivation, social development, and well‐being. Am Psychol. 2000;55(1):68‐78. 11392867 10.1037//0003-066x.55.1.68

[75] Ryan R , Patrick H , Deci E , Williams G . Facilitating health behavior change and its maintenance: interventions based on Self‐Determination Theory. Eur. Health Psychol. 2008;10:2‐5.

[76] Krebs P , Prochaska JO , Rossi JS . A meta‐analysis of computer‐tailored interventions for health behavior change. Prev Med. 2010;51(3‐4):214‐221. 20558196 10.1016/j.ypmed.2010.06.004 PMC2939185

[77] Smit E , Linn A , Weert J . Taking online computer‐tailoring forward. The potential of tailoring the message frame and delivery mode of online health behaviour change interventions. Eur J Health Psychol. 2015;17:25‐31.

[78] Morrison LG . Theory‐based strategies for enhancing the impact and usage of digital health behaviour change interventions: a review. Digit Health. 2015;1:205520761559533. 10.1177/2055207615595335 PMC5999061 29942544

[79] Perski O , Short CE . Acceptability of digital health interventions: embracing the complexity. Transl Behav Med. 2021;11(7):1473‐1480. 33963864 10.1093/tbm/ibab048 PMC8320880

[80] The British Standards Institution . ISO 9241‐11: Ergonomics of Human–System Interaction—Part 11: Usability: Definitions and Concepts. 2nd ed. BSI Standards Ltd; 2018.

[81] Sharif F , Rahman A , Tonner E , et al. Can technology optimise the pre‐operative pathway for elective hip and knee replacement surgery: a qualitative study. Perioper Med. 2020;9(1):33. 10.1186/s13741-020-00166-0 PMC7667783 33292556

[82] Keogh‐Brown M , Bachmann M , Shepstone L , et al. Contamination in trials of educational interventions. Health Technol Assess (Rockv). 2007;11(43):iii, ix‐107. 10.3310/hta11430 17935683

[83] Krug S . Don't Make Me Think, Revisited: A Common Sense Approach to Web Usability. 3rd ed. New Riders; 2014.

[84] Muller I , Santer M , Morrison L , et al. Combining qualitative research with PPI: reflections on using the person‐based approach for developing behavioural interventions. Res Involv Engagem. 2019;5(1):34. 31807316 10.1186/s40900-019-0169-8 PMC6857167

[85] Pawson R . The Science of Evaluation: A Realist Manifesto. SAGE Publications Ltd; 2013. 10.4135/9781473913820

