# Supplementary material for: Supporting patients to prepare for total knee replacement: Evidence‐, theory‐ and person‐based development of a ‘Virtual Knee School’ digital intervention
Source: Health Expect. 2023 Aug 22;26(6):2549–70. doi: 10.1111/hex.13855 (PMC10632615; doi:10.1111/hex.13855)
Supplement: Supplementary file 1 — Supporting information. [file HEX-26--s003.docx]

**Supplementary File 1: Phase 4 methods supporting information**

# Table S1: Virtual Knee School education section intervention planning table excerpt

| **Page(s)** | **VKS feature** | **Importance level^a^** | | | **Time-consuming to develop** | **Priority** | **Include in prototype** |
| --- | --- | --- | --- | --- | --- | --- | --- |
|  |  | **1** | **2** | **3** |  |  |  |
| Managing concerns during your recovery | Text covering how to lower the risks of having issues after TKR surgery, including wound care | – | NICE; VIR (1.14) | – | No | Should have | Yes |
|  | Traffic light system checklist that:   - includes complications of TKR surgery and common issues that do not need to cause alarm - explains how to organise help if complications occur | – | VIR (1.14, 1.15) | IR (1.33); BF (Ed3) | No | Should have | Yes |
| Getting up and about | Brief videos of patient models demonstrating how to use:   - a walking stick - one and two crutches - a walking frame | VGP (3) | PPI | IR (1.8, 1.34); BF (W3, Ed2, Ed3) | Yes | Must have | Yes |
|  | Text covering post-operative mobility, including:   - the role of mobilising in rehabilitation following TKR surgery - the role of mobilising in lowering the risks of TKR surgery - key points about mobilising safely | – | VIR (1.14, 1.20) | IR (1.34) | No | Should have | Yes |
|  | Accordion content covering:   - returning to a normal walking pattern - how to stand up, sit down and perform bed transfers | – | – | IR (1.8, 1.34); BF (W3, Ed3) | No | Could have | Yes |
|  | PDF booklets covering how to use walking aids | – | PPI | IR (1.8, 1.34, 2.5.3, 2.6) | Yes | Could have | No |
|  | Photographs of a patient model getting up with one foot in front of the other and getting up with their feet in line | – | – | BF (Ed2) | Yes | Would like | No |
|  | Photograph of a patient model getting on and off a bed | – | – | BF (Ed2) | Yes | Would like | No |
|  | Brief video of a patient model getting up with one foot in front of the other and getting up with their feet in line | – | – | BF (W3, Ed2, Ed3) | Yes | Would like | No |
|  | Brief video of a patient model getting on and off a bed | – | – | BF (W3, Ed2, Ed3) | Yes | Would like | No |

^a^ Table 2 in the main paper provides the meaning of the codes.

Abbreviations: PDF, Portable Document Format; TKR, total knee replacement; VKS, Virtual Knee School

# Virtual Knee School exercise programme design overview

The Virtual Knee School (VKS) exercise programme was designed using a multi-step process.

1. A list of target exercise types to include in the VKS exercise programme was identified from the final set of recommendations developed in the Phase 1 modified Delphi study (1). A table was created to document the target exercise types, exercises that could be classified as each exercise type, and considerations for deciding which exercise types/exercises to include in the VKS prototype (Table S2).
2. The findings from Table S2 and research team discussions were used to develop proposed exercise categories for the VKS exercise programme and a prioritised list of candidate exercises for each category (Table S3).
3. The findings from Table S3 and research team discussions were used to develop a table summarising the proposed exercise categories and exercises to be included in the VKS. This was refined based on discussions with two Project Advisory Group (PAG) Patient and Public Involvement (PPI) members to create the finalised list of VKS exercise categories and exercises (Table S4).
4. A proposed delivery format for the VKS exercise programme was developed based on the final set of recommendations developed in the Delphi study (1) and research team discussions (Table S5). The delivery approaches were discussed with two PAG PPI members, who felt no changes were needed.

## Table S2: Target exercise types

| **Target exercise type (Phase 1b modified Delphi study item number (1))^a^** | **Exercises classified as the exercise type in randomised studies of pre-operative interventions included in the Phase 1a rapid review (2)^bc^** | **Exercises identified from additional sources [source]^b^** | **Considerations for including the exercise type/potential exercises in the VKS prototype** |
| --- | --- | --- | --- |
| Leg strengthening exercises (Item 3.1) | - Squats with elastic resistance (3-5) - Dynamic stepping exercise (6) - Hip extension (with a strength training machine or with elastic resistance) (4, 5, 7) - Hip flexion with elastic resistance (4, 5) - Hip abduction (with a strength training machine or with elastic resistance, on an even or an uneven surface) (4, 5, 7-11) - Hip adduction (with a strength training machine or with elastic resistance, on an even or an uneven surface) (4, 7, 10, 11) - Seated leg press (with a strength training machine) (7-9) - Knee extension (with elastic resistance, with a strength training machine or with no external resistance, in sitting) (4-12) - Isometric quadriceps contraction in full extension using a rolled towel under the knee in supine (6) - Hamstring flexion/leg curl (with elastic resistance, with a strength training machine or with no external resistance, in prone/side lying or in sitting) (3-11) - Unspecified quadriceps strengthening (13) - Ankle dorsiflexion with elastic resistance (3-5) - Ankle plantar flexion with elastic resistance (3-5) | - Dynamic joint movements and dynamic body weight movements, including step-ups and calf raises [RR (8, 9)] - *“Leg lifts with rolled towel under knee”* [DC] - *“Terminal knee extensions”* [DC] - *“Straight leg raises”* [PPI] | - Exercises that require elastic resistance or a strength training machine are inconsistent with VGP-5 due to requiring specific equipment. However, many of the exercises listed as being performed with elastic resistance or a strength training machine can be performed in alternative ways that do not require equipment. - *‘Dynamic joint movements and dynamic body movements’* can include multiple different exercises. - *‘Dynamic stepping exercise’* / *‘Step-ups’* can also be classified as a *‘Functional movement exercise’*, *‘Cardiovascular exercise’* and *‘Training on steps’*. |
| Leg flexibility exercises (Item 3.3) | - Gluteal stretch (14) - Hip extensor stretch (3) - Hip flexor stretch (3) - Hip adductor stretch (14) - Knee extensor/quadriceps stretch (in sitting) (3, 6, 7, 14) - Knee flexor/hamstring stretch (in sitting) (3, 6, 7, 14) - Ankle flexors/gastrocnemius stretch (7, 14) | - Gluteal stretch [RR (4, 5)] - Hip stretch [RR (4, 5)] - Hip abductors stretch [RR (8, 9)] - Knee extensors stretch [RR (8, 9)] - Knee flexors/hamstring stretch [RR (4, 5, 8, 9)] - Ankle plantar flexors/calf stretch [RR (4, 5, 8, 9)] - Unspecified lower limb mobility exercises and stretches (one exercise is shown but was used for patients with hip osteoarthritis only) [RR (10, 11)] | None of note. |
| Balance exercises (Item 3.6) | - Double leg stance on an unstable device (8, 9) - Single leg stance (on an unstable device, hard floor or balance mat, with or without support, with eyes open or closed) (8, 9, 14) - Slide step forward/backward on a hard floor or balance mat, with or without support, with eyes open or closed (14) - Step forward/backward on a hard floor or balance mat, with or without support, with eyes open or closed (14) - Squats on a hard floor or balance mat, with or without support, with eyes open or closed (14) | - *“Balancing on Bosu”* [DC] - *“Heel to toe walking”* [RR (14), PPI] | - Exercises that require a balance device/mat are inconsistent with VGP-5 due to requiring specific equipment. - *‘Slide step forward/backward’* and *‘Step forward/backward’* can also be classified as *‘Leg strengthening exercises’* and *‘Functional technique exercises’*. - *‘Squats’* can also be classified as a *‘Leg strengthening exercise’*. |
| Functional movement exercises (Item 3.7) | - Forward step ups, with or without hand support or bar bells (10, 11) - Chair stands, with or without hand support, with feet parallel or with one foot forward (10, 11) | - Transfer training (bed, vehicle and toilet transfers) [DC (two panellists)] | - Transfer training will be addressed in the education section of the VKS. - Forward step-ups can also be classified as a *‘Leg strengthening exercise’*, *‘Cardiovascular exercise’* and *‘Training on steps’*. - Chair stands can also be classified as a *‘Leg strengthening exercise’* and *‘Cardiovascular exercise’*. |
| Functional technique exercises (Item 3.8) | - Slide-exercise forward-backward on an even or uneven surface, with flexion/extension of the weight-bearing knee (10, 11) - Slide-exercise sideways on an even or uneven surface, with flexion/extension of the weight-bearing knee (10, 11) - Forward lunge, with hand support if required (10, 11) - Sideways lunge, with hand support if required (10, 11) | - Walking forward and backwards in front of a mirror [RR (10, 11)] | - Walking forward and backwards in front of a mirror is inconsistent with VGP-5 due to requiring specific equipment. - Slide-exercise forward-backward and slide-exercise sideways can also be classified as *‘Leg strengthening exercises’* and *‘Balance exercises’*. - Forward lunge and sideways lunge can also be classified as *‘Leg strengthening exercises’* and *‘Balance exercises’*. |
| Cardiovascular exercises (Item 3.11) | None – cardiovascular exercises were added to Round 1 based on project team discussions during the pilot testing process. | - Unweighted leg joint movements [RR (3)] - Walking (fast-paced) [RR (4, 5, 14)] - Ergometer cycling (hand or leg) [RR (7-11)] | - *‘Unweighted leg joint movements’* can include multiple different exercises. - *‘Ergometer cycling’* is inconsistent with VGP-5 due to requiring specific equipment. - *‘Walking’* and *‘Ergometer cycling (leg)’* can also be classified as *‘Leg strengthening exercises’*. |
| Core control exercises (Item 3.12) | - Pelvic lifts with gym ball (10, 11) - Sit-ups with gym ball (10, 11) | None | - Exercises that require a gym ball are inconsistent with VGP-5 due to requiring specific equipment. However, both core control exercises listed can be performed without a gym ball. |
| Walking practice with walking aids (Item 3.13) | None – this exercise type was included in the Round 1 survey based on a study exploring patients’ and health professionals’ views of pre-operative interventions (15) | None | - This exercise type is inconsistent with VGP-5 due to requiring specific equipment. However, *‘Walking’* alone is consistent with VKS guiding principles. - Guidance on obtaining and using walking aids will be included in the VKS education section. |
| Training on steps (Item 3.14) | - Forwards step-ups (3-5) - Lateral step-ups (3-5) | None | - *‘Forward step-ups’* and *‘Lateral step-ups’* can also be classified as *‘Leg strengthening exercises’*, *‘Functional movement exercises’* and *‘Cardiovascular exercises’* |
| Practicing post-operative exercises (Item 3.15) | None – this exercise type was added to Round 2 based on content analysis of the Round 1 free-text responses. | - Knee flexion/extension in sitting with a sliding device (e.g. plastic bag or skateboard) under the foot [PPI, DC] | - All the exercise listed are appropriate to perform post-operatively, although some are not appropriate in the early post-operative phase. - *‘Knee flexion/extension in sitting with a sliding device’* can also be classified as a *‘Leg flexibility exercise’.* |

^a^ All the exercise types included in the final set of recommendations developed in the Phase 1b modified Delphi study were considered target exercise types for inclusion in the VKS.

^b^ Exercises were only identified from studies that were identified prior to the rapid review search updates and reported a statistically significant difference in favour of the intervention group for at least one outcome at one or more follow-up time points.

^c^ The exercise classifications were based on the primary study authors’ descriptions. Details in brackets were specified in at least one, but not all, of the studies listed.

Abbreviations: DC, free-text comment provided by a panellist in the Phase 1b modified Delphi study; PPI, free-text comment provided by a Patient and Public Involvement representative during pilot testing of Round 1 of the Phase 1b modified Delphi study; RR, exercise identified from the warm-up or cool-down of a randomised study of a pre-operative intervention(s) included in the Phase 1a rapid review; VGP-5, Virtual Knee School guiding principle 5; VKS, Virtual Knee School

## Table S3: Proposed Virtual Knee School exercise categories and candidate exercises

| **Proposed exercise category^a^** | **Prioritised candidate exercises^ab^** | **Explanation** |
| --- | --- | --- |
| Aerobic fitness | 1. Walking or marching on the spot *(standing)* 2. Step-ups (forward step-ups) *(standing)* 3. Seated marching *(sitting)* 4. Sideways step-ups (lateral step-ups) *(standing)* | - This exercise category focuses primarily on *‘Cardiovascular exercises’* and *‘Training on steps’*. - *‘Marching on the spot’* was added as an alternative to walking to account for users with limited space. - *‘Seated marching’* was selected as an appropriate *‘Unweighted leg joint movements’* exercise. - It was decided to name this category *‘Aerobic fitness’* because the included exercises will raise users’ heart rates. |
| Knee strength and endurance | 1. Chair stands *(sitting/standing)* 2. Knee straightening (knee extensions) *(sitting)* 3. Straight leg raise *(crook lying)* 4. Mini squats (squats) *(standing)* 5. Leg lifts with a rolled towel under the knee *(long sitting)* | - This exercise category focuses primarily on *‘Leg strengthening exercises’* and *‘Functional movement exercises’*. - Improving knee extensor muscle strength is a key target of TKR prehabilitation (16). Therefore, it was agreed it was important to separate *‘Leg strengthening exercises’* into two separate sections, one focusing solely on knee extensor exercises and one focusing on hip and ankle exercises. Correspondingly, it was decided not to include *‘Hamstring flexion/leg curl’* in this category to help ensure that users perform at least one knee extensor strengthening exercise. - Given the exercises listed may improve both muscle strength and endurance, it was decided to name this category *‘Knee strength and endurance’*. |
| Hip and ankle strength and endurance | 1. Heel raises (calf raises) *(standing)* 2. Sideways leg lifts (hip abduction) *(standing/side lying)* 3. Backwards leg lifts (hip extension) *(standing/prone)* 4. Knee lifts (hip flexion) *(standing)* 5. Toe lifts (ankle dorsiflexion) *(standing)* 6. Towel squeezes (hip adduction) *(sitting)* | - This exercise category focuses on *‘Leg strengthening exercises’*. - Exercises listed as being performed with elastic resistance or a strength training machine in the rapid review studies have been adapted so that they can be performed without requiring specific equipment. - As above, it was decided to name this category ‘*Hip and ankle strength and endurance’* because the exercises listed aim to improve muscle strength and endurance. |
| Balance and stability | 1. Standing on one leg (single leg stance) *(standing)* 2. Hip lifts (pelvic lifts) *(crook lying)* 3. Step forwards/backwards (forward lunge) *(standing)* 4. Heel to toe walking *(standing)* 5. Slide-exercise sideways *(standing)* 6. Slide-exercise forwards/backwards (slide step forward/backward) *(standing)* 7. Step sideways (sideways lunge) *(standing)* 8. Sit-ups *(crook lying)* | - This exercise group focuses primarily on *‘Balance exercises’*, *‘Functional technique exercises’* and *‘Core stability exercises’.* - *‘Step forwards/backwards’* is considered to include *‘Forward lunge’* as the size of the step forwards can be varied. - *‘Slide step forwards/backwards’* *and ‘Slide-exercise forwards/backwards’* are listed as different exercise types in the target exercise types table but are considered the same exercise. |
| Leg stretching and flexibility | 1. Thigh stretch (knee extensor stretch) *(standing/side lying)* 2. Hamstring stretch *(standing/sitting)* 3. Knee bending/straightening (knee flexions/extensions in sitting with a sliding device) *(sitting)* 4. Calf stretch *(standing)* 5. Buttock stretch (gluteal stretch) *(long sitting)* 6. Knee to chest stretch (hip extensor stretch) *(crook lying)* 7. Inner thigh stretch (hip adductor stretch) *(standing/sitting)* 8. Hip stretch *(standing)* | - This exercise category focuses on *‘Leg flexibility exercises’* and *‘Practicing post-operative exercises’.* |

^a^ The exercise categories/candidate exercises covered all the target exercise types except for *‘Walking practice with walking aids’* because requiring specific equipment is inconsistent with Virtual Knee School (VKS) guiding principle 5 and the VKS education section provides guidance on using walking aids.

^b^ Bracketed plain text indicates the original description of the exercise, which was amended for clarity. Bracketed text in italics indicates the exercise position(s). It was provisionally decided to include the top three candidate exercises from each category in the VKS exercise programme; therefore, the prioritisation aimed to ensure that the top three exercises in each category varied in difficulty and included at least one non-weight bearing exercise to address VKS guiding principle 5, and that the top three exercises from all five categories combined covered all the target exercise types except *for ‘Walking practice with walking aids’*.

Abbreviations: TKR, total knee replacement

##

## Table S4: Finalised Virtual Knee School exercise categories and exercises

| **Exercise category** | **Exercises** |
| --- | --- |
| 1. Aerobic fitness | Seated marching  Walking on the spot  Step-ups |
| 1. Knee strength and endurance | Straight leg raise  Knee straightening  Sit to stand |
| 1. Hip and ankle strength and endurance | Sideways leg lifts  Backwards leg lifts  Heel raises |
| 1. Balance and stability | Hip lifts  Standing on one leg  Step forwards and backwards |
| 1. Leg flexibility | Thigh stretch  Hamstring stretch  Calf stretch |

## Table S5: Virtual Knee School exercise programme delivery approaches

| **Delivery category** | **Phase 1b modified Delphi study final recommendation (item number(s) (1))** | **Addressed in the VKS** | **Explanation** |
| --- | --- | --- | --- |
| Delivery mode | Be delivered using a combination of more than one format, including supervised exercise sessions, unsupervised exercise sessions and a booklet or other written format (4.1.2; 4.1.3; 4.1.5; 4.2) | Partly | The exercise programme was provided directly on the VKS prototype through text/videos with captions and as a PDF booklet that users could download. |
|  | Provide an opportunity for peer support (4.11) | No | An online discussion forum would require moderation. This would be inconsistent with VGP-1. |
|  | Include goal setting (4.12) | Yes | The VKS prototype included a goal-setting feature (details below). |
| Intensity | Include exercises which are low to moderate intensity (4.4.2) | Yes | The exercise instructions recommended starting at a low level and slowly building up to a medium level. |
|  | Be progressive (4.6) | Yes | The exercise instructions encouraged users to progress by:   - increasing the number of exercise sessions they perform per week; - increasing the intensity of the exercises; - increasing the number of exercises they perform per session. |
| Schedule | Involve exercise sessions which last a minimum of fifteen minutes each (4.7) | Yes | The exercise instructions recommended selecting at least one exercise from each category (five exercises in total) and performing three sets of 30 seconds of each exercise, with a 30-second rest after each set. |
|  | Involve a minimum of two exercise sessions per week (4.8) | Yes | The exercise instructions encouraged users to perform at least two exercise sessions per week. |
|  | Ideally be performed for a minimum of six weeks (4.9) | Partly | The exercise instructions encouraged users to start the programme as soon as possible. A specific timeframe was not provided because patients remain on the TKR waiting list for varying lengths of time. |
| Tailoring | Be tailored according to each patient’s individual needs and ability (4.5; 4.10) | Yes | The content and delivery of the exercise programme were self-tailored because users could choose from a range of exercises and adapt the intensity and schedule to meet their individual needs and ability. |

Abbreviations: PDF, Portable Document Format; TKR, total knee replacement; VGP-1, Virtual Knee School guiding principle 1; VKS, Virtual Knee School

# Think-aloud interview recruitment approaches

As detailed in the main paper, participants were recruited via a National Health Service Teaching Hospital and word of mouth. The following recruitment approaches were also employed with the aim of facilitating the recruitment of patients who were male and/or from a Black, Asian, or other minority ethnic group.

1. A PAG PPI member shared a WhatsApp message with contacts in her communities.
2. Recruitment adverts were posted on Twitter and Facebook.
3. Two local community networks that work with people from underserved groups were approached.

The WhatsApp message and social media adverts included details of specific eligibility criteria, for example to highlight that people from Black, Asian, or other minority ethnic groups were particularly welcome.

No participants were recruited in response to the WhatsApp message or social media adverts, and neither of the community networks approached were willing to share the recruitment adverts.

# Think-aloud interview topic guide

The think-aloud interview topic guide provided below was developed based on the Phase 4 objectives and an example think-aloud topic guide for person-based approach intervention development studies (17). Neither of the two PAG PPI members who were invited to review the topic guide suggested any changes. The lead researcher pilot tested the topic guide with another member of the research team prior to the first interview. No modifications were made to the topic guide during the study.

**Development of a Virtual Knee School, Phase 4, think-aloud interview topic guide**

IRAS 262809; version 3.0, dated 04 Aug 2021

*The following topic guide may be modified during the data collection phase so that themes identified in earlier interviews can be explored in later interviews. Each participant will complete two interviews. This topic guide will be used for both interviews. Consent will have been obtained online prior to the participant’s first interview.*

**Interview Introduction**

*The interviewer should complete all the following actions prior to commencing the interview*

1. Review the information provided in the Participant Information Sheet, including:

- Aim of the study
- Participant can withdraw at any time
- Interview will be recorded with an encrypted mobile phone, laptop and/or secure video conferencing tool
- Confidentiality

1. Explain the process for the interview, including:

- Participant should say what they are thinking out loud as they work through the Virtual Knee School
- Interviewer may ask prompt questions
- Interview is not a test and there are no right or wrong answers
- Interviewer cannot answer questions during the interview, but can discuss them at the end
- Interviewer may ask the participant to access specific information/sections/pages of the Virtual Knee School
- Once the participant has finished working through the Virtual Knee School, the interviewer will ask questions about the participant’s overall views of the Virtual Knee School

1. Reiterate the Virtual Knee School is still in development and is not fully ready to be used
2. Offer the participant an opportunity to ask questions

**Think-aloud Prompts**

*The interviewer may ask the participant any of the following prompt questions as the participant works through the Virtual Knee School. Each prompt may be used multiple times, if appropriate. The prompts may be adapted/expanded for clarity.*

1. Can you tell me what you think about the <<insert website content>> on this page?
2. Can you tell me how you feel about using <<insert digital feature/activity/tool>>?
3. Can you tell me what you like about <<insert website content/digital feature/activity/tool>>?
4. Can you tell me what you DON’T like about <<insert website content/digital feature/activity/tool>>?
5. Can you tell me why you selected that?
6. Can you tell me about your overall views of this page?
7. Can you tell me what you are thinking at the moment?
8. Can you explain that a bit more?

**Post-Think-aloud Prompts**

1. What are your overall views of the Virtual Knee School?
2. Can you tell me about anything you particularly liked about the Virtual Knee School?
3. Can you tell me about anything you particularly DIDN’T like about the Virtual Knee School?
4. How do you think the Virtual Knee School could be improved?
5. Is there anything else you would like to add?

**Interview closure**

*The interviewer should complete all the following actions after the interview is completed*

1. Thank the participant for taking part in the interview
2. If the interview was the participant’s first interview, confirm the plan for their second interview
3. Offer the participant an opportunity to ask questions
4. Complete the travel expenses form if the interview took place at [recruitment site]

# Table S6: Table of changes main section excerpt

| **Page/ aspect** | **Positive comment [participant pseudonym]** | **Negative comment [participant pseudonym]** | **Suggested change** | **Reason for change^a^** | **Time-consuming to implement** | **Priority MoSCoW** | **Change agreed** | **Date change implemented^b^** |
| --- | --- | --- | --- | --- | --- | --- | --- | --- |
| Homepage |  | Does not feel it is clear from the homepage that the website has three key sections. [Glen] | Add text to the homepage to explain that the website has three key sections. | VGP (1) BEH (web-based) EAS | No | Must have | Agreed 28/10/2021 | 28/10/2021 |
|  |  |  | Change the *'About the Virtual Knee School'* text button to a picture button next to the buttons to the other key sections (so that there are three picture buttons corresponding with the three key sections). | VGP (1) BEH (web-based) | No | Must have | Agreed 28/10/2021 | 28/10/2021 |
|  | Likes colour, layout and “*less writing”* (compared to the *'About the Virtual Knee School'* page). Feels it is *“very clear but not overwhelming”.* [Ella]  Feels the page layout is clear. [Arthur] Feels the page layout is *“easy to use, clear, and not confusing.”* [Haaniya] Feels the page layout *“works, and it’s nice, ‘cause it’s very visual, so it makes it more appealing doesn’t it? It’s not just all text.”* [Naomi] Feels the homepage layout is *“helpful”.* [Zuri] |  |  |  |  |  |  |  |

^a^ The meanings of the codes are provided in Table 4 in the main text.

^b^ An additional column called *‘Notes’* was included in each Excel sheet but is not shown due to space limitations. The *‘Notes’* column was used to document any key points related to the potential change, such as comments from the research team discussions and the time requirements for changes that would have to be made by the Frank team rather than the research team.

Abbreviations: MoSCoW, *‘Must have, Should have, Could have, Would like’* model (18, 19)

# References

1. Anderson AM, Comer C, Smith TO, Drew BT, Pandit H, Antcliff D, et al. Consensus on pre-operative total knee replacement education and prehabilitation recommendations: a UK-based modified Delphi study. BMC Musculoskelet Disord. 2021;22(1):352.

2. Anderson AM, Drew BT, Antcliff D, Redmond AC, Comer C, Smith TO, et al. Content and delivery of pre-operative interventions for patients undergoing total knee replacement: a rapid review. Syst Rev. 2022;11(1):184.

3. Brown K, Topp R, Brosky JA, Lajoie AS. Prehabilitation and quality of life three months after total knee arthroplasty: a pilot study. Percept Mot Skills. 2012;115(3):765-74.

4. Swank AM, Kachelman JB, Bibeau W, Quesada PM, Nyland J, Malkani A, et al. Prehabilitation before total knee arthroplasty increases strength and function in older adults with severe osteoarthritis. J Strength Cond Res. 2011;25(2):318-25.

5. Topp R, Swank AM, Quesada PM, Nyland J, Malkani A. The effect of prehabilitation exercise on strength and functioning after total knee arthroplasty. PM R. 2009;1(8):729-35.

6. Matassi F, Duerinckx J, Vandenneucker H, Bellemans J. Range of motion after total knee arthroplasty: the effect of a preoperative home exercise program. Knee Surg Sports Traumatol Arthrosc. 2014;22(3):703-9.

7. Skoffer B, Maribo T, Mechlenburg I, Hansen PM, Søballe K, Dalgas U, et al. Efficacy of Preoperative Progressive Resistance Training on Postoperative Outcomes in Patients Undergoing Total Knee Arthroplasty. Arthritis Care Res (Hoboken). 2016;68(9):1239-51.

8. Calatayud J, Casana J, Ezzatvar Y, Jakobsen MD, Sundstrup E, Andersen LL. High-intensity preoperative training improves physical and functional recovery in the early post-operative periods after total knee arthroplasty: a randomized controlled trial. Knee Surg Sports Traumatol Arthrosc. 2017;25(9):2864-72.

9. Casaña J, Calatayud J, Ezzatvar Y, Vinstrup J, Benitez J, Andersen LL. Preoperative high-intensity strength training improves postural control after TKA: randomized-controlled trial. Knee Surg Sports Traumatol Arthrosc. 2019;27(4):1057-66.

10. Villadsen A, Overgaard S, Holsgaard-Larsen A, Christensen R, Roos EM. Immediate efficacy of neuromuscular exercise in patients with severe osteoarthritis of the hip or knee: a secondary analysis from a randomized controlled trial. J Rheumatol. 2014;41(7):1385-94.

11. Villadsen A, Overgaard S, Holsgaard-Larsen A, Christensen R, Roos EM. Postoperative effects of neuromuscular exercise prior to hip or knee arthroplasty: a randomised controlled trial. Ann Rheum Dis. 2014;73(6):1130-7.

12. Tungtrongjit Y, Weingkum P, Saunkool P. The effect of preoperative quadriceps exercise on functional outcome after total knee arthroplasty. J Med Assoc Thai. 2012;95 (Suppl 10):S58-66.

13. Jahic D, Omerovic D, Tanovic AT, Dzankovic F, Campara MT. The Effect of Prehabilitation on Postoperative Outcome in Patients Following Primary Total Knee Arthroplasty. Med Arch. 2018;72(6):439-43.

14. Gstoettner M, Raschner C, Dirnberger E, Leimser H, Krismer M. Preoperative proprioceptive training in patients with total knee arthroplasty. Knee. 2011;18(4):265-70.

15. Westby MD, Marshall DA, Jones CA. Development of quality indicators for hip and knee arthroplasty rehabilitation. Osteoarthritis Cartilage. 2018;26(3):370-82.

16. Husted RS, Juhl C, Troelsen A, Thorborg K, Kallemose T, Rathleff MS, et al. The relationship between prescribed pre-operative knee-extensor exercise dosage and effect on knee-extensor strength prior to and following total knee arthroplasty: a systematic review and meta-regression analysis of randomized controlled trials. Osteoarthritis Cartilage. 2020;28(11):1412-26.

17. PBA: Person-Based Approach. Useful Resources. 2019. <https://www.personbasedapproach.org/resources.html>. Accessed 07 Sep 2019.

18. Bradbury K, Watts S, Arden-Close E, Yardley L, Lewith G. Developing digital interventions: a methodological guide. Evid Based Complement Alternat Med. 2014;2014:561320.

19. Kuhn J. Decrypting the MoSCoW analysis 2009;5(44).
