# Supplementary material for: Supporting patients to prepare for total knee replacement: Evidence‐, theory‐ and person‐based development of a ‘Virtual Knee School’ digital intervention
Source: Health Expect. 2023 Aug 22;26(6):2549–70. doi: 10.1111/hex.13855 (PMC10632615; doi:10.1111/hex.13855)
Supplement: Supplementary file 2 — Supporting information. [file HEX-26--s002.docx]

**Supplementary File 2: Phase 3 findings supporting information**

# Groups of considerations used to develop the Virtual Knee School guiding principles

Six groups of considerations related to the intended Virtual Knee School (VKS) users’ characteristics, context and needs were identified from the sources in Table S7. Each group of considerations was used to develop a guiding principle as detailed in the main paper.

## Table S7: Key sources used during the theoretical modelling

| **Code** | **Source** |
| --- | --- |
| PPI-C | Patient and Public Involvement consultations held during the project planning. |
| RR (study citation) | Findings from the Phase 1a rapid review studies (1). |
| DR (item number) | Final set of recommendations developed in the Phase 1b modified Delphi study (2). |
| DC | Free-text comments provided by patients and/or professionals in the Phase 1b modified Delphi study (2). |
| FG | Focus group findings from the Phase 2 qualitative descriptive study. |

## Pre-operative total knee replacement (TKR) intervention provision and digital delivery

There are substantial discrepancies in current United Kingdom (UK) pre-operative total knee replacement (TKR) intervention provision [PPI-C; FG]. Some patients do not receive sufficient pre-operative TKR education and prehabilitation support or do not receive it long enough before their surgery [PPI-C; DC; FG]. These inadequacies may encourage patients to engage with web-based pre-operative TKR care [FG]. However, some patients may not be able to access websites [PPI-C; DC]. Even patients who can access websites may be reluctant to use them [FG]. Some patients have concerns about the reliability of websites and/or the detail/duration of website interactions [PPI-C; FG]. Furthermore, patients’ experience of using digital tools and preferences for digital features vary widely [FG].

## Pre-operative TKR education concerns

Although some patients want to find out what happens during TKR surgery, others may be concerned about receiving information about the TKR surgical procedure, particularly due to the risk of seeing graphic details of surgery [RR (3); DC; FG]. In addition, patients may have concerns about hearing *“horror stories”* of TKR [FG]. Hearing such stories may impair patients’ ability to learn [RR (4)]. Patients may also be concerned about making comparisons with other patients [FG]. On the other hand, for some patients, a desire to find out about other patients’ experiences of TKR may be a facilitator to engagement with pre-operative TKR education [RR (5); FG].

## Pre-operative TKR education preferences and needs

Patients’ preferences for pre-operative TKR education vary widely, with some patients wanting to receive as much pre-operative information as possible, whilst others do not want to receive detailed information [RR (3); FG]. Patients’ learning styles also differ [RR (6)]. In addition, patients may have low literacy and/or face language barriers [RR (6); DC; FG]. Correspondingly, some patients need simple information, but others find large volumes of simple information frustrating [FG]. Some patients value educational videos, especially of practical tasks such as how to use walking aids [FG]. Key topics that patients want information on include understanding what to expect, pain management and rehabilitation [PPI-C; RR (3, 5, 7-9); DR (1.12; 1.14–1.20); DC; FG].

## Pre-operative TKR exercise misconceptions and motivating factors

Some patients may be concerned that exercising will cause further knee damage [PPI-C; FG]. Patients may also believe that pre-operative exercises are not important/beneficial [RR (10); DC]. This belief may be reinforced by health professionals [DC; FG]. Conversely, patients may be particularly motivated to perform pre-operative TKR exercises by the belief that doing so will improve their post-operative recovery and a sense of personal responsibility for their own recovery [RR (5); DC; FG]. Patients may also be motivated to engage with pre-operative exercises by setting goals/receiving tailored feedback and monitoring their exercise completion [FG]. Correspondingly, goal setting was identified as an important element of pre-operative TKR exercise programmes during the Phase 1b modified Delphi study [DR (4.12)].

## Pre-operative TKR exercise preferences and needs

Patients listed for TKR surgery typically have severe knee signs/symptoms, which can prevent them from exercising [DC; FG]. Patients’ engagement with pre-operative exercises may also be limited by a lack of guidance, being busy with other commitments/distractions and additional personal preferences/circumstances such as a dislike of certain exercise types, having other health issues and not being able to access specific equipment/facilities [PPI-C; RR (10); FG]. Patients’ preferences for exercise videos versus exercise animations vary [FG]. Some patients particularly value exercise videos with real-life models, but videos may have a negative impact if patients cannot relate to the models [FG].

## Pre-operative healthy lifestyle change motivating factors, needs and preferences

Patients may be motivated to make pre-operative healthy lifestyle changes by the belief that doing so will improve their post-operative recovery, a sense of personal responsibility for their own recovery and strategies such as self-monitoring and reflection [RR (5, 11, 12); FG]. As for pre-operative exercise, patients’ engagement with healthy lifestyle changes may be limited by a lack of guidance [FG]. However, credible sources of healthy lifestyle guidance that account for individuals’ differing needs/preferences are already available [FG].

# Behavioural analysis tables overview

Behavioural analysis tables were created for each behaviour targeted by the VKS (tables S8–S11). Sets of barriers and facilitators to the target behaviours and potential VKS features that could address the barriers and facilitators were identified from the sources listed in Table S7. Extra features were added based on project team discussions. All the features were mapped to components of the Capability, Opportunity, Motivation, Behaviour (COM-B) model of behaviour (13), intervention functions from the Behaviour Change Wheel (BCW) (13), and behaviour change techniques from the Behaviour Change Technique Taxonomy v1 (BCTTv1) (14).

## Table S8: Behavioural analysis table for engagement with pre-operative TKR care in a web-based format

| **Set of barriers/*facilitators* to the target behaviour**  **[barrier/facilitator/VKS feature source(s)^a^]** | **Potential VKS feature(s) that could address the barriers/facilitators** | **Target component(s) (BCW)** | **Intervention function(s) (BCW)** | **Behaviour change technique(s) (BCTTv1)** |
| --- | --- | --- | --- | --- |
| W1. Limited experience of using digital tools/low digital literacy  [RR (15); FG] | Simple navigation, including menu pages with links to other sections/pages. | Physical opportunity | Environmental restructuring | 12.1 Restructuring the physical environment |
|  | Introductory section that provides clear instructions about how to use the VKS and emphasises that it is easy to use, even for people who have limited experience of using digital tools.  *‘Common questions’* and *‘Help’* pages available to assist users with using the VKS. | Psychological capability  Reflective motivation | Education  Persuasion | 4.1 Instruction on how to perform the behaviour  15.1 Verbal persuasion about capability |
| W2. Reluctance to use digital technologies  Concerns about the reliability of websites  Concerns about receiving conflicting information  [PPI-C, RR (7); FG] | Introductory section that:   - highlights the potential benefits of using the VKS, including that it supports users to prepare for TKR surgery, understand what to expect and perform a pre-operative exercise programme, which could help improve users’ post-operative recovery; - explains that the VKS adds to the guidance patients get from their own care team and patients should always follow guidance from their own care teams; - highlights that the VKS is NIHR-funded (and so linked to the NHS) and has been developed by experts based on evidence and feedback from patients; - includes names, biographies and/or photographs of team members; - acknowledges that some people are reluctant to use digital technologies and explains that the VKS provides documents that users can download and print out where appropriate. | Psychological capability  Reflective motivation | Education  Persuasion | 5.1 Information about health consequences  5.6 Information about emotional consequences  9.1 Credible source |
|  | Documents that users can download and print out. | Physical opportunity | Environmental restructuring | 12.5 Adding objects to the environment |
| W3. Concerns about the detail/duration of website interactions  [PPI-C; RR (15); FG] | Brief videos.  Digital tools with quick simple recording.  Menu bar and search box to enable rapid navigation.  Accordion content and a small number of links that provide further information. | Physical opportunity | Environmental restructuring | 12.1 Restructuring the physical environment |
| W4. Reluctance to use a goal setting feature  [FG] | Explanation about the potential benefits of goal setting. | Reflective motivation | Persuasion | 5.1 Information about health consequences  5.6 Information about emotional consequences |
| W5. *Family member support*  [DR 2.9; FG] | Introductory section that highlights family and friends can assist with using the VKS. | Social opportunity | Enablement | 3.1 Social support (unspecified)  12.2 Restructuring the social environment |

^a^ Codes for the sources are provided in Table S7.

Abbreviations: BCTTv1, Behaviour Change Technique Taxonomy version 1 (14); BCW, Behaviour Change Wheel (13); NHS, National Health Service; NIHR, National Institute for Health and Care Research; TKR, total knee replacement; VKS, Virtual Knee School; W, set of barriers/facilitators to engagement with pre-operative TKR care in a web-based format

## Table S9: Behavioural analysis table for engagement with pre-operative TKR education

| **Set of barriers/*facilitators* to the target behaviour**  **[barrier/facilitator/VKS feature source(s)^a^]** | **Potential VKS feature(s) that could address the barriers/facilitators** | **Target component(s) (BCW)** | **Intervention function(s) (BCW)** | **Behaviour change technique(s) (BCTTv1)** |
| --- | --- | --- | --- | --- |
| Ed1. Short length of time between being listed for TKR surgery and undergoing TKR surgery  Receiving information immediately after the decision to undergo TKR surgery is made/too far in advance of surgery  [RR (7, 16); FG] | Information section that allows all content to be accessed rapidly during any session. | Physical opportunity | Environmental restructuring | 12.1 Restructuring the physical environment |
| Ed2. Low literacy  Language barriers  *Information presented using methods that address patients’ varying health literacy, language abilities and learning styles*  [RR (6, 15); DC; FG] | Provide information using simple language, pictures and videos where appropriate.  Include a glossary to explain medical terms patients may come across.  Provide an option to change the VKS language. | Physical opportunity | Environmental restructuring | 12.1 Restructuring the physical environment |
| Ed3. Reluctance to receive detailed pre-operative information  Large volume of information  *Desire for detailed information about preparing for TKR surgery and what to expect*  [PPI-C; RR (3, 5, 7, 8, 12, 15, 16); DR 1.4–1.20, 1.22–1.25, 1.30, 1.32 – 1.35; DC; FG] | Introductory section that explains the VKS supports users to prepare for TKR surgery and understand what to expect, which can help their recovery after surgery. | Reflective motivation | Persuasion | 5.1 Information about health consequences  5.6 Information about emotional consequences |
|  | Information about how to prepare for TKR surgery and what to expect provided through accessible and engaging formats, including:   - brief glossary of medical terms and a more detailed glossary available as a PDF document; - accordion content and a small number of links that provide further information; - checklists e.g. a “traffic light system” checklist about complications and a checklist about home preparations; - videos, including of real people performing practical tasks such as using walking aids, getting in/out of a car, getting up from a fall and going round the supermarket. | Physical opportunity | Environmental restructuring | 12.1 Restructuring the physical environment |
| Ed4. Concerns about receiving information about the TKR surgical procedure and/or seeing graphic details of TKR surgery  *Desire to understand what happens during TKR surgery*  [RR (3); DR (1.13); DC; FG] | Introductory section that explains the VKS will not show graphic details of TKR surgery. | Psychological capability  Reflective motivation | Education | 5.6 Information about emotional consequences |
|  | Brief text and animation about the TKR surgical procedure that does not show any graphic details of surgery. | Physical opportunity | Environmental restructuring | 12.1 Restructuring the physical environment |
| Ed5. Concerns about finding out about “horror stories” of TKR surgery  Concerns about making comparisons with other patients’ experiences of TKR surgery  *Desire to find out about other patients’ experiences of TKR surgery*  [RR (4, 5); DR 2.4; DC; FG] | Introductory section that:   - explains that the VKS provides examples of other patients’ experiences of TKR surgery to help users understand what to expect; - explains that everyone’s preparations and recovery are different. | Psychological capability  Reflective motivation | Education  Persuasion | 5.6 Information about emotional consequences |
|  | Information about TKR surgery provided through appropriately moderated patient stories, which are unlikely to be interpreted as “horror stories”. | Social opportunity  Reflective motivation | Persuasion  Modelling | 6.2 Social comparison  6.3 Information about others’ approval  9.1 Credible source |

^a^ Codes for the sources are provided in Table S7.

Abbreviations: BCTTv1, Behaviour Change Technique Taxonomy version 1 (14); BCW, Behaviour Change Wheel (13); Ed, set of barriers/facilitators to engagement with pre-operative TKR education; PDF, Portable Document Format; TKR, total knee replacement; VKS, Virtual Knee School

## Table S10: Behavioural analysis table for engagement with a pre-operative TKR exercise programme

| **Set of barriers/*facilitators* to the target behaviour**  **[barrier/facilitator/VKS feature source(s)^a^]** | **Potential VKS feature(s) that could address the barriers/facilitators** | **Target component(s) (BCW)** | **Intervention function(s) (BCW)** | **Behaviour change technique(s) (BCTTv1)** |
| --- | --- | --- | --- | --- |
| Ex1. Knee signs and symptoms  [PPI-C; DR (2.4, 4.4.2, 4.6); DC; FG] | Flexible exercise programme that includes:   - non-weight bearing exercises; - low to moderate intensity exercises; - guidance on how to select exercises, including starting at a relatively easy level and then gradually progressing. | Physical capability  Psychological capability  Physical opportunity | Education  Training  Environmental restructuring | 1.2 Problem solving  4.1 Instruction on how to perform a behaviour  5.1 Information about health consequences  8.1 Behavioural practice/rehearsal  8.7 Graded tasks  12.1 Restructuring the physical environment |
|  | Patient stories modelling how other patients have successfully performed a pre-operative exercise programme despite severe knee signs and symptoms. | Psychological capability  Social opportunity  Reflective motivation | Education  Persuasion  Modelling | 5.1 Information about health consequences  6.2 Social comparison  6.3 Information about others’ approval  9.1 Credible source |
| Ex2. Concern that exercising will cause further knee damage  [PPI-C; DR (2.4); FG] | Information reassuring users that performing pre-operative exercises is safe for people with severe knee arthritis. | Psychological capability  Reflective motivation | Education  Persuasion | 5.1 Information about health consequences |
|  | Patient stories modelling how other patients have successfully performed a pre-operative exercise programme despite having severe knee arthritis. | Psychological capability  Social opportunity  Reflective motivation | Education  Persuasion  Modelling | 5.1 Information about health consequences  6.2 Social comparison  6.3 Information about others’ approval  9.1 Credible source |
| Ex3. Being busy with other commitments/distractions Forgetting to exercise  [PPI-C; RR (10); FG] | Suggestion about setting exercise reminders, for example on a mobile phone.  Optional automated email reminders prompting users to perform exercises, with flexible timing. | Physical opportunity | Environmental restructuring | 7.1 Prompts/cues |
|  | Information about the benefits of integrating exercise into daily routines (habit formation) and suggestions about how to do so. | Psychological capability  Reflective motivation  Automatic motivation | Education  Persuasion  Training | 4.1 Instruction on how to perform a behaviour  8.1 Behavioural practice/rehearsal  8.3 Habit formation |
| Ex4. Other health issues  [PPI-C; DC; FG] | Brief information about exercising with specific health conditions. | Psychological capability | Education | 5.1 Information about health consequences |
|  | Flexible exercise programme that includes:   - non-weight bearing exercises; - low to moderate intensity exercises. | Physical capability Psychological capability  Physical opportunity | Education  Training  Environmental restructuring | 1.2 Problem solving  4.1 Instruction on how to perform a behaviour  5.1 Information about health consequences  12.1 Restructuring the physical environment |
|  | Guidance on seeking health professional advice about other health issues that may present a barrier to exercise. | Social opportunity | Enablement | 3.2 Social support (unspecified)  9.1 Credible source |
| Ex5. Lack of access to specific equipment or facilities  [FG] | Flexible exercise programme that includes exercises that do not require non-household equipment or facilities. | Physical opportunity | Environmental restructuring | 12.1 Restructuring the physical environment |
| Ex6. Belief that pre-operative exercises are not important/beneficial  *Beliefs about the benefits of pre-operative exercise, including on post-operative recovery*  *Sense of personal responsibility for own recovery*  [PPI-C; (5, 10); DR (1.4, 1.5, 2.4); DC; RR FG] | Introductory and exercise sections that explain the potential benefits of pre-operative exercise, including for post-operative recovery.  Explanations about the benefits of specific exercises/reasons for specific exercises.  Guidance to support users identify their reasons for wanting to exercise pre-operatively. | Psychological capability  Reflective motivation | Education  Persuasion | 1.2 Problem solving  5.1 Information about health consequences  5.6 Information about emotional consequences |
|  | Information explaining that experts agree pre-operative exercise is beneficial (based on the NICE guidelines). | Reflective motivation | Persuasion | 6.3 Information about others’ approval  9.1 Credible source |
|  | Patient stories modelling how other patients have performed and benefitted a pre-operative exercise programme. | Psychological capability  Social opportunity  Reflective motivation | Education  Persuasion  Modelling | 5.1 Information about health consequences  5.6 Information about emotional consequences  6.2 Social comparison  6.3 Information about others’ approval  9.1 Credible source |
| Ex7. Lack of guidance on performing a pre-operative exercise programme  *Guidance on performing a pre-operative exercise programme*  [DC; FG] | Flexible exercise programme that includes:   - guidance on how to select and progress exercises; - videos of relatable patient representatives demonstrating how to perform exercises, with appropriate audio explanations of the exercises. | Psychological capability  Social opportunity | Education  Training  Modelling | 1.2 Problem solving  4.1 Instruction on how to perform a behaviour  5.1 Information about health consequences  6.1 Demonstration of the behaviour  6.2 Social comparison  8.1 Behavioural practice/rehearsal  8.7 Graded tasks |
|  | Tips on exercising from peers | Psychological capability  Social opportunity  Reflective motivation | Education  Modelling | 4.1 Instruction on how to perform the behaviour  6.2 Social comparison  6.3 Information about others’ approval  9.1 Credible source |
| Ex8. Dislike of certain exercise types or formats  *Preference for certain exercise types or formats*  [FG] | Explanations about the benefits of specific exercises/reasons for specific exercises. | Psychological capability  Reflective motivation | Education  Persuasion | 5.1 Information about health consequences  5.6 Information about emotional consequences |
|  | Guidance to support users to identify and perform other types of exercise they enjoy alongside the VKS exercise programme. | Psychological capability  Reflective motivation | Education  Persuasion | 1.2 Problem solving  4.1 Instruction on how to perform a behaviour  5.6 Information about emotional consequences |
| Ex9. Setting exercise goals and not meeting them  *Setting exercise goals, reviewing exercise goals and receiving feedback about exercise goals*  [PPI-C; DR (1.6, 4.12); DC; FG] | Exercise goal setting, review and feedback feature that includes:   - information about goal setting, including its benefits and how to set achievable goals; - suggestions about how to adapt goals if they are not met; - encouraging feedback; - goal setting and recording sheet that users can download and print out. | Psychological capability  Physical opportunity  Reflective motivation | Education  Persuasion  Environmental restructuring  Enablement | 1.1 Goal setting (behaviour)  1.2 Problem solving  1.4 Action planning  1.5 Review behaviour goal(s)  1.6 Discrepancy between current behaviour and goal  2.2 Feedback on behaviour  2.3 Self-monitoring of behaviour  5.1 Information about health consequences  10.4 Social reward  12.5 Adding objects to the environment |
| Ex10. *Monitoring exercise completion*  [FG] | Guidance on monitoring exercise completion.  Exercise diary that users can download and print out.  Private online personal exercise diary. | Physical opportunity  Reflective motivation | Environmental restructuring  Enablement | 2.3 Self-monitoring of behaviour  12.5 Adding objects to the environment |
| Ex11. *Family member support*  [RR (17)] | Explanation that some patients find it helpful to exercise with family members or friends. | Social opportunity | Enablement | 3.1 Social support (unspecified)  12.2 Restructuring the social environment |

^a^ Codes for the sources are provided in Table S7.

Abbreviations: BCTTv1, Behaviour Change Technique Taxonomy version 1 (14); BCW, Behaviour Change Wheel (13); Ex, set of barriers/facilitators to engagement with a pre-operative TKR exercise programme; NICE, National Institute for Health and Care Excellence; VKS, Virtual Knee School

## Table S11: Behavioural analysis table for engagement with healthy lifestyle changes

| **Set of barriers/*facilitators* to the target behaviour**  **[barrier/facilitator/VKS feature source(s)^a^]** | **Potential VKS feature(s) that could address the barriers/facilitators** | **Target component(s) (BCW)** | **Intervention function(s) (BCW)** | **Behaviour change technique(s) (BCTTv1)** |
| --- | --- | --- | --- | --- |
| **Healthy lifestyle change: increase physical activity and reduce sedentary behaviour^b^** | | | | |
| HL1. Knee signs and symptoms  Fatigue  Poor physical fitness  [PPI-C; RR (10, 11); DR (2.4); FG] | Information about the potential benefits of non-weight bearing activities and examples of non-weight bearing activities. | Physical capability  Reflective motivation | Education | 5.1 Information about health consequences |
|  | Information about the potential benefits of activity pacing and guidance on how to pace activities.  Activity planning sheet that users can download and print out. | Physical capability  Physical opportunity  Psychological capability  Reflective motivation | Education  Training  Environmental restructuring | 1.2 Problem solving  1.4 Action planning  4.1 Instruction on how to perform a behaviour  5.1 Information about health consequences  8.7 Graded tasks  12.5 Adding objects to the environment |
|  | Information about the potential benefits of using walking aids and videos of real people demonstrating how to use walking aids. | Physical capability  Psychological capability  Social opportunity  Reflective motivation | Education  Training  Modelling  Enablement | 4.1 Instruction on how to perform a behaviour  5.1 Information about health consequences  6.1 Demonstration of the behaviour  6.2 Social comparison  12.6 Body changes (assistive aids) |
|  | Information about the potential benefits of using analgesics and the importance of taking them regularly as advised by the user’s care team. | Physical capability  Reflective motivation | Education | 5.1 Information about health consequences |
|  | Information about the potential benefits of using cushioned soles/insoles. | Physical capability  Reflective motivation | Education | 5.1 Information about health consequences |
|  | Patient stories modelling how other patients have successfully increased their activity levels/reduced their sedentary behaviour despite severe knee signs and symptoms, fatigue and poor physical fitness. | Psychological capability  Social opportunity  Reflective motivation | Education  Persuasion  Modelling | 5.1 Information about health consequences  6.2 Social comparison  6.3 Information about others’ approval  9.1 Credible source |
| HL2. Forgetfulness  [RR (11)] | Guidance on setting activity reminders, for example on a mobile phone. | Physical opportunity | Environmental restructuring | 7.1 Prompts/cues |
|  | Information about the benefits of habit formation and suggestions about how to make being more active/less sedentary a habit. | Psychological capability  Reflective motivation  Automatic motivation | Education  Persuasion  Training | 4.1 Instruction on how to perform a behaviour  8.1 Behavioural practice/rehearsal  8.3 Habit formation |
| HL3. Fear of falling  [RR (11)] | Guidance to support users to choose activities that users are likely to be able to do with minimal risk of falling. | Psychological capability  Reflective motivation | Education  Persuasion | 1.2 Problem solving  5.1 Information about health consequences |
| HL4. Other health issues  [RR (11)] | Guidance on seeking health professional advice about other health issues that may present a barrier to being more active/less sedentary. | Social opportunity | Enablement | 3.2 Social support (unspecified)  9.1 Credible source |
| HL5. Social and environmental circumstances (including lack of time, social responsibilities/ commitments, going on holiday and finding it difficult to do physical activities in the evening or in certain weather conditions)  [RR (11)] | Guidance to support users to identify barriers to being more active/less sedentary and strategies for addressing the barriers. | Psychological capability  Reflective motivation | Education | 1.2 Problem solving  1.4 Action planning |
| HL6. *Beliefs about the benefits of being more active/less sedentary*  *Sense of achievement*  [RR (11)] | Explanations about the benefits of and reasons for being more active/less sedentary.  Guidance to support users identify their reasons for wanting to be more active/less sedentary. | Psychological capability  Reflective motivation | Education  Persuasion | 1.2 Problem solving  5.1 Information about health consequences  5.6 Information about emotional consequences |
|  | Patient stories modelling how other patients have benefitted from being more active/less sedentary. | Psychological capability  Social opportunity  Reflective motivation | Education  Persuasion  Modelling | 5.1 Information about health consequences  5.6 Information about emotional consequences  6.2 Social comparison  6.3 Information about others’ approval  9.1 Credible source |
| HL7. *Enjoyment of certain activities*  [RR (11)] | Guidance to support users to identify/perform physical activities they enjoy. | Psychological capability  Reflective motivation | Education  Persuasion | 1.2 Problem solving  4.1 Instruction on how to perform a behaviour  5.6 Information about emotional consequences |
| HL8. *Self-reflection on sedentary time/activity levels*  [RR (11)] | Guidance to support users to monitor and reflect on their current activity levels/sedentary behaviour, identify barriers to being more active/less sedentary and identify strategies for addressing the barriers. | Psychological capability  Reflective motivation | Education  Enablement | 1.2 Problem solving  1.4 Action planning  2.3 Self-monitoring of behaviour |
|  | Sedentary behaviour/physical activity screening feature that provides personalised feedback. | Reflective motivation | Persuasion | 2.2 Feedback on behaviour  5.1 Information about health consequences |
| HL9. Activity goals perceived as futile or pointless  Setting too challenging goals  *Setting challenging but achievable goals*  [RR (11)] | General guidance on goal setting, including setting challenging but achievable goals and adapting goals if they are not met. | Psychological capability | Education | 1.1 Goal setting (behaviour)  1.2 Problem solving  1.4 Action planning |
|  | Exercise goal setting, review and feedback feature that includes:   - information about goal setting, including its benefits and how to set achievable goals; - an option to set a goal about another type of physical activity, in addition to the VKS exercise programme; - suggestions about how to adapt goals if they are not met; - encouraging feedback; - goal setting and recording sheet that users can download and print out. | Psychological capability  Physical opportunity  Reflective motivation | Education  Persuasion  Environmental restructuring  Enablement | 1.1 Goal setting (behaviour)  1.2 Problem solving  1.4 Action planning  1.5 Review behaviour goal(s)  1.6 Discrepancy between current behaviour and goal  2.2 Feedback on behaviour  2.3 Self-monitoring of behaviour  5.1 Information about health consequences  10.4 Social reward  12.5 Adding objects to the environment |
| HL10. Issues with monitoring activity using a pedometer  *Monitoring physical activity*  [RR (11); FG] | Guidance on self-monitoring activity levels/sedentary behaviour, including signposting to an activity tracking app that users can download on a mobile phone. | Reflective motivation | Enablement | 2.3 Self-monitoring of behaviour |
| **Healthy lifestyle change: improve weight management and diet** | | | | |
| HL11. Lack of guidance on weight management  [DR (1.27); DC; FG] | Guidance on weight management strategies. | Psychological capability | Education | 4.1 Instruction on how to perform the behaviour  5.1 Information about health consequences |
|  | Signposting to credible websites that provide weight management advice. | Psychological capability | Education | 5.1 Information about health consequences  9.1 Credible source |
| HL12. Other health issues or lifestyle choices  [FG] | Signposting to credible websites that provide weight management advice that accounts for other health issues or lifestyle choices. | Psychological capability | Education | 5.1 Information about health consequences  9.1 Credible source |
|  | Guidance on seeking health professional advice about other health issues that may present a barrier to weight management. | Social opportunity | Enablement | 3.2 Social support (unspecified)  9.1 Credible source |
| HL13. Difficulty adhering to diets (including due to a tendency to overeat)  [FG] | Guidance to support users to identify barriers to adhering to diets and strategies for addressing the barriers. | Psychological capability  Reflective motivation | Education | 1.2 Problem solving  1.4 Action planning |
| HL14. *Beliefs about the benefits of healthy eating and weight management, including on post-operative recovery*  *Sense of personal responsibility for own recovery*  [ RR (5); DR (1.4); FG] | Explanations about the benefits of/reasons for healthy eating and weight management, including on post-operative recovery.  Guidance to support users identify their reasons for wanting to lose weight. | Psychological capability  Reflective motivation | Education  Persuasion | 1.2 Problem solving  5.1 Information about health consequences  5.6 Information about emotional consequences |
|  | Patient stories modelling how other patients have benefitted from managing their weight. | Psychological capability  Social opportunity  Reflective motivation | Education  Persuasion  Modelling | 5.1 Information about health consequences  5.6 Information about emotional consequences  6.2 Social comparison  6.3 Information about others’ approval  9.1 Credible source |
| HL15. *Monitoring eating habits*  [FG] | Guidance on self-monitoring eating habits, including signposting to an app that can be used to track eating habits. | Reflective motivation | Enablement | 2.3 Self-monitoring of behaviour |
| **Healthy lifestyle change: reduce alcohol consumption** | | | | |
| HL16. Environmental circumstances (including going on holiday pre-operatively)  [FG] | Guidance to support users to identify barriers to reducing their alcohol consumption and strategies for addressing the barriers. | Psychological capability  Reflective motivation | Education | 1.2 Problem solving  1.4 Action planning |
| HL17. Older age/being retired, potentially leading boredom; a tendency to be less risk-averse; being more affluent; feeling lonely; considering themselves too old to change their drinking habits and not wanting to be ‘educated’ about alcohol use.  *Older age, potentially leading to greater motivation to look after their health and a tendency to be more open and honest about their drinking habits*  [RR (12)] | Guidance to support users to identify barriers to reducing their alcohol consumption and strategies for addressing the barriers. | Psychological capability  Reflective motivation | Education | 1.2 Problem solving  1.4 Action planning |
| HL18. Not perceiving excess alcohol consumption as an issue/not perceiving themselves a ‘risky’ or ‘problem’ drinker/being defensive about drinking habits  ‘Risky drinking’ sounding appealing  Alcohol consumption being a sensitive subject  Not understanding the terms ‘standard drink’ and/or ‘unit’  *Being open and honest about drinking habits (linked to being older, a patient and/or male)*  [RR (12)] | Information about alcohol consumption guidelines and the risks of excess alcohol consumption/benefits of reducing alcohol consumption presented clearly and sensitively, including:   - not using the terms ‘risky drinking’, ‘risky drinker’ or ‘problem drinker’; - using terms such as ‘pint of beer’ and ‘glass of wine’.   Infographic explaining standard drinks. | Psychological capability  Physical opportunity  Reflective motivation | Education  Persuasion | 5.1 Information about health consequences  5.6 Information about emotional consequences  12.1 Restructuring the physical environment |
| HL19. Not understanding the impact of pre-operative alcohol consumption on outcomes of TKR surgery  *Desire to have TKR surgery*  *Beliefs about the benefits of reducing alcohol consumption, including on post-operative recovery and weight*  *Sense of obligation to prepare for TKR surgery and help ensure it goes well*  [RR (12)] | Information about the risks of excess alcohol consumption/ benefits of reducing alcohol consumption, including on post-operative recovery and weight.  Guidance to support users to identify their reasons for wanting to reduce their alcohol consumption. | Psychological capability  Reflective motivation | Education  Persuasion | 1.2 Problem solving  5.1 Information about health consequences  5.6 Information about emotional consequences |
|  | Patient stories modelling how other patients have benefitted from reducing their pre-operative alcohol consumption. | Psychological capability  Social opportunity  Reflective motivation | Education  Persuasion  Modelling | 5.1 Information about health consequences  5.6 Information about emotional consequences  6.2 Social comparison  6.3 Information about others’ approval  9.1 Credible source |
| HL20. Not realising how much alcohol they are consuming  *Identifying and reflecting on their alcohol consumption*  [RR (12)] | Guidance to support the user to monitor and reflect on their current alcohol consumption, identify barriers to reducing their alcohol consumption and identify strategies for addressing the barriers. | Psychological capability  Reflective motivation | Education  Enablement | 1.2 Problem solving  1.4 Action planning  2.3 Self-monitoring of behaviour |
|  | Alcohol consumption screening feature that provides personalised feedback. | Reflective motivation | Persuasion | 2.2 Feedback on behaviour  5.1 Information about health consequences |

^a^ Codes for the sources are provided in Table S7.

^b^ Only includes details related to increasing physical activity in general rather than engaging in a pre-operative TKR exercise programme.

Abbreviations: BCTTv1, Behaviour Change Technique Taxonomy version 1 (14); BCW, Behaviour Change Wheel (13); HL, set of barriers/facilitators to engagement with healthy lifestyle changes; TKR, total knee replacement; VKS, Virtual Knee School

# Table S12: Behaviour change techniques employed in the potential Virtual Knee School features

| **Cluster^a^** | **Behaviour change technique^a^** |
| --- | --- |
| 1. Goals and planning | 1.1 Goal setting (behaviour) |
|  | 1.2 Problem solving |
|  | 1.4 Action planning |
|  | 1.5 Review behaviour goal(s) |
|  | 1.6 Discrepancy between current behaviour and goal |
| 2. Feedback and monitoring | 2.2 Feedback on behaviour |
|  | 2.3 Self-monitoring of behaviour |
| 3. Social support | 3.1 Social support (unspecified) |
| 4. Shaping knowledge | 4.1 Instruction on how to perform a behaviour |
| 5. Natural consequences | 5.1 Information about health consequences |
|  | 5.6 Information about emotional consequences |
| 1. Comparison of behaviour | 6.1 Demonstration of the behaviour |
|  | 6.2 Social comparison |
|  | 6.3 Information about others’ approval |
| 7. Associations | 7.1 Prompts/cues |
| 8. Repetition and substitution | 8.1 Behavioural practice/rehearsal |
|  | 8.3 Habit formation |
|  | 8.7 Graded tasks |
| 9. Comparison of outcomes | 9.1 Credible source |
| 10. Reward and threat | 10.4 Social reward |
| 12. Antecedents | 12.1 Restructuring the physical environment |
|  | 12.2 Restructuring the social environment |
|  | 12.5 Adding objects to the environment |
|  | 12.6 Body changes (assistive aids) |
| 15. Self-belief | 15.1 Verbal persuasion about capability |

^a^ Clusters and behaviour change techniques are from the Behaviour Change Technique Taxonomy (v1) (14).

# Table S13: Additional behaviour change techniques identified in the review by Safari et al. (18)

| **Cluster^a^** | **Behaviour change technique^a^** |
| --- | --- |
| 1. Goals and planning | 1.7 Review outcome goals |
| 2. Feedback and monitoring | 2.4 Self-monitoring of outcome(s) of behaviour |
|  | 2.6 Biofeedback |
| 3. Social support | 3.2 Social support (practical) |
|  | 3.3 Social support (emotional) |
| 4. Shaping knowledge | 4.2 Information about antecedents |
| 5. Natural consequences | 5.4 Monitoring of emotional consequences |
|  | 5.5 Anticipated regret |
| 9. Comparison of outcomes | 9.2 Pros and cons |
| 11. Regulation | 11.2 Reduce negative emotions |
| 12. Antecedents | 12.4 Distraction |
| 15. Self-belief | 15.4 Self-talk |
| 16. Covert learning | 16.2 Imaginary reward |
|  | 16.3 Vicarious consequences |

^a^ Clusters and behaviour change techniques are from the Behaviour Change Technique Taxonomy (v1) (14). The behaviour change techniques were identified in a systematic review of digital-based structured osteoarthritis self-management programmes by Safari et al. (18) but were not employed in any of the potential VKS features.

# References

1. Anderson AM, Drew BT, Antcliff D, Redmond AC, Comer C, Smith TO, et al. Content and delivery of pre-operative interventions for patients undergoing total knee replacement: a rapid review. Syst Rev. 2022;11(1):184

2. Anderson AM, Comer C, Smith TO, Drew BT, Pandit H, Antcliff D, et al. Consensus on pre-operative total knee replacement education and prehabilitation recommendations: a UK-based modified Delphi study. BMC Musculoskelet Disord. 2021;22(1):352.

3. Berg U, Berg M, Rolfson O, Erichsen-Andersson A. Fast-track program of elective joint replacement in hip and knee-patients' experiences of the clinical pathway and care process. J Orthop Surg Res. 2019;14(1):186.

4. Høvik LH, Aglen B, Husby VS. Patient experience with early discharge after total knee arthroplasty: a focus group study. Scand J Caring Sci. 2018;32(2):833-42.

5. Goldsmith LJ, Suryaprakash N, Randall E, Shum J, MacDonald V, Sawatzky R, et al. The importance of informational, clinical and personal support in patient experience with total knee replacement: a qualitative investigation. BMC Musculoskelet Disord. 2017;18(1):127.

6. Causey-Upton R, Howell DM, Kitzman PH, Custer MG, Dressler EV. Orthopaedic Nurses' Perceptions of Preoperative Education for Total Knee Replacement. Orthop Nurs. 2020;39(4):227-37.

7. Judge A, Carr A, Price A, Garriga C, Cooper C, Prieto-Alhambra D, et al. The impact of the enhanced recovery pathway and other factors on outcomes and costs following hip and knee replacement: routine data study. Southampton (UK): NIHR Journals Library. 2020.

8. Smith DH, Kuntz J, DeBar L, Mesa J, Yang X, Boardman D, et al. A qualitative study to develop materials educating patients about opioid use before and after total hip or total knee arthroplasty. J Opioid Manag. 2018;14(3):183-90.

9. Huber EO, Bastiaenen CH, Bischoff-Ferrari HA, Meichtry A, de Bie RA. Development of the knee osteoarthritis patient education questionnaire: a new measure for evaluating preoperative patient education programmes for patients undergoing total knee replacement. Swiss Med Wkly. 2015;145:w14210.

10. Bin Sheeha B, Williams A, Johnson DS, Granat M, Jones R. Patients' experiences and satisfaction at one year following primary total knee arthroplasty: A focus-group discussion. Musculoskeletal Care. 2020;18(4):434-49.

11. Aunger JA, Greaves CJ, Davis ET, Asamane EA, Whittaker AC, Greig CA. A novel behavioural INTErvention to REduce Sitting Time in older adults undergoing orthopaedic surgery (INTEREST): results of a randomised-controlled feasibility study. Aging Clin Exp Res. 2020;32(12):2565-85.

12. Snowden C, Lynch E, Avery L, Haighton C, Howel D, Mamasoula V, et al. Preoperative behavioural intervention to reduce drinking before elective orthopaedic surgery: the PRE-OP BIRDS feasibility RCT. Health Technol Assess. 2020;24(12):1-176.

13. Michie S, van Stralen MM, West R. The behaviour change wheel: A new method for characterising and designing behaviour change interventions. Implement Sci. 2011;6(1):42.

14. Michie S, Richardson M, Johnston M, Abraham C, Francis J, Hardeman W, et al. The Behavior Change Technique Taxonomy (v1) of 93 hierarchically clustered techniques: building an international consensus for the reporting of behavior change interventions. Ann Behav Med. 2013;46(1):81-95.

15. Sharif F, Rahman A, Tonner E, Ahmed H, Haq I, Abbass R, et al. Can technology optimise the pre-operative pathway for elective hip and knee replacement surgery: a qualitative study. Perioper Med (Lond). 2020;9(1):33.

16. Specht K, Kjaersgaard‐Andersen P, Pedersen BD. Patient experience in fast-track hip and knee arthroplasty - a qualitative study. J Clin Nurs. 2016;25(5-6):836-45.

17. Lucas B, Cox C, Perry L, Bridges J. Pre-operative preparation of patients for total knee replacement: An action research study. Int J Orthop Trauma Nurs. 2013;17(2):79-90.

18. Safari R, Jackson J, Sheffield D. Digital Self-Management Interventions for People With Osteoarthritis: Systematic Review With Meta-Analysis. J Med Internet Res. 2020;22(7):e15365.
