# Supplementary material for: Supporting patients to prepare for total knee replacement: Evidence‐, theory‐ and person‐based development of a ‘Virtual Knee School’ digital intervention
Source: Health Expect. 2023 Aug 22;26(6):2549–70. doi: 10.1111/hex.13855 (PMC10632615; doi:10.1111/hex.13855)
Supplement: Supplementary file 3 — Supporting information. [file HEX-26--s001.docx]

**Supplementary File 3: Phase 4 findings supporting information**

# Table S14: Virtual Knee School prototype summary

| **Section** | **Page(s)^a^** | **Level** | **Key features^b^** |
| --- | --- | --- | --- |
| Login | *‘Sign up to the Virtual Knee School’* | N/A | Sign up process that involved entering an email address, password and participant identification number and completing a CAPTCHA verification. |
|  | *‘Login’* | N/A | Login process that involved entering an email address and password.  Reset password option. |
| Main | *‘Welcome’* (homepage) | 1 | Brief text summarising the VKS purpose.  Picture buttons to the other three level 1 pages. |
|  | *‘Help’* | N/A | Text and accordion content explaining how to use the VKS/overcome problems users may encounter when using the VKS. |
|  | *‘Contact us’* | N/A | Text providing the VKS email address. |
|  | Footer pages | N/A | Privacy and cookies policy, accessibility statement, links to other helpful websites. |
|  | All pages | N/A | Header with *‘Help’* and *‘Log out’* buttons.  Footer containing links to the footer pages, University of Leeds terms of use and the user’s most viewed pages.  Meganav and search box.  Breadcrumb trail (not shown on the homepage).  *‘Print this page’* button (not shown on the homepage).  Accessibility toolbar that allows users to change the VKS language, text size and contrast (automatically open but can be opened and closed by selecting the toolbar header). |
| Introductory | *‘About the Virtual Knee School’* (introductory menu) | 1 | Picture buttons to both level 2 introductory pages.  Text and an image explaining how to use the accessibility toolbar.  Welcome video designed to address key barriers to engagement with the VKS and its target behaviours.  Text navigation instructions. |
|  | *‘Virtual Knee School development and team’* | 2 | Text providing a brief overview of the VKS development.  PDF of the Phase 1b modified Delphi study final recommendations (1).  Names, photographs and brief biographies of key research team members.  Names of the additional research team members and the PAG PPI members. |
|  | *‘Common questions’* | 2 | Accordion content with answers to questions about the VKS and how to use it. |
| Education | *‘Information for your operation’* (education menu) | 1 | Picture buttons to all three level 2 education pages. |
|  | *‘What to expect’* | 2 | Picture buttons to all seven level 3 expectations subpages. |
|  | Seven expectations subpages | 3 | Text/accordion content covering TKR surgery; what to expect before, during and after the hospital stay; risks of TKR surgery; a brief list of medical terms; and patients’ knee replacement stories.  Knee joint anatomy image, PDF list of medical terms and five educational videos. |
|  | *‘Preparing for your operation’* | 2 | Picture buttons to all seven level 3 preparing subpages. |
|  | Seven preparing subpages | 3 | Text/accordion content covering managing knee pain; healthy lifestyle changes; goal setting; walking aids and other equipment; making practical preparations; return to work planning; and patients’ preparation stories.  PDF exercise goal-setting/recording sheet, two educational videos and 10 walking aid videos. |
|  | *‘Recovering from your operation’* | 2 | Picture buttons to all six level 3 recovering subpages. |
|  | Six recovering subpages | 3 | Text/accordion content covering strategies for improving post-operative recovery; managing concerns (including a traffic light checklist); post-operative mobilisation, returning to usual activities and travelling; and patients’ recovery stories.  Three educational videos and 10 walking aid videos. |
| Exercise | *‘Your exercise plan’* (exercise menu) | 1 | Picture buttons to all five level 2 exercise pages.  Text covering benefits of exercising pre-operatively, guidance about the VKS exercise programme and essential safety information. |
|  | *‘About the Virtual Knee School exercise plan’* | 2 | Text covering key questions and answers about the VKS exercise programme, including potential concerns and safety considerations. |
|  | *‘Tips for sticking to your exercise plan’* | 2 | Text covering goal setting, self-monitoring exercise completion, habit formation, identifying reasons for wanting to exercise and setting exercise reminders.  PDF exercise goal-setting/recording sheet and PDF exercise diary. |
|  | *‘Patients’ exercise stories’* | 2 | Two patient stories modelling how patients have successfully overcome barriers to and benefited from exercising pre-operatively. |
|  | *‘Your exercise goals’* | 2 | Text explaining the benefits of goal setting.  PDF exercise goal-setting/recording sheet and PDF exercise diary.  Buttons to set new goals and view current goals.  Dated list of goals with options to edit goals and review goals/edit review. |
|  | Four exercise goal subpages | 3 | Goal setting form that includes two VKS exercise goals (required) and a personal exercise goal (optional).  Text summarising the goals set.  Goal review form with *‘Yes’*, *‘Partly’* and *‘No’* options for each goal set.  Goal feedback that is personalised based on the goal review form responses, with tips on how the user could adapt/progress their goals. |
|  | *‘Carry out an exercise session’* | 2 | Text advising users to view the *‘Your exercise plan’* and *‘About the Virtual Knee School’ exercise plan’* pages before performing an exercise session, with hyperlinks to both pages.  Text providing guidance on how to perform an exercise session.  Fifteen exercise videos organised in five categories, with text explaining the benefits of each exercise category.  PDF exercise booklet. |

^a^ Text in italics in single quotation marks is the page name displayed in the website banner.

^b^ All static images were accompanied by PDF documents and all videos were accompanied by transcripts/a booklet for accessibility.

Abbreviations: CAPTCHA, Completely Automated Public Turing test to tell Computers and Humans Apart; PAG, Project Advisory Group; PDF, Portable Document Format; PPI, Patient and Public Involvement; TKR, total knee replacement; VKS, Virtual Knee School

# Figure S1: Think-aloud interview participant flow chart

**Community recruitment: contacts received (n=6)**

Twitter (n=0)

Facebook (n=0)

WhatsApp (n=0)

Word-of-mouth (n=4)

Unknown (n=2)

**NHS recruitment: patients approached (n=29)**

Posted information pack (n=15)

Orthopaedic consultant clinic (n=2)

Pre-assessment clinic (n=12)

**Not screened (n=11)**

Did not have time to speak to the researcher for screening (n=1)

Did not respond to posted information pack (n=9)

Did not respond to email follow-up (n=1)

**Screened (n=24)**

**Excluded (n=14)**

Not meeting eligibility criteria (n=2)

Not meeting purposive selection criteria (n=4)

Did not return contact form (n=5)

Declined participation (n=2)

Sufficient participants already recruited (n=1)

**Invited to participate (n=10)**

**Declined participation (n=1)**

Work commitments (n=1)

**Consented (n=9)**

**Participated in first interview (n=9)**

**Withdrew (n=2)**

Increased anxiety (n=1)

Serious health problems (n=1)

**Participated in second interview (n=7)**

NHS, National Health Service

# Table S15: Think-aloud interview participant characteristics

|  | **Number of participants (%) (n=9)** |
| --- | --- |
| **Age (years)** | |
| 40–49 | 1 (11) |
| 50–59 | 1 (11) |
| 60–69 | 3 (33) |
| 70–79 | 3 (33) |
| 80-89 | 1 (11) |
| **Gender** | |
| Female | 3 (33) |
| Male | 6 (67) |
| **Experience of TKR** | |
| Pre | 3 (33) |
| Post | 4 (44) |
| Pre, post | 2 (22) |
| **Confidence in using the Internet** | |
| Very confident | 2 (22) |
| Confident | 3 (33) |
| Neither confident nor unconfident | 2 (22) |
| Unconfident | 2 (22) |
| **Indication for TKR^a^** | |
| Osteoarthritis | 9 (82) |
| Rheumatoid arthritis and osteoarthritis | 2 (18) |
| **Location of TKR^a^** | |
| NHS hospital | 10 (91) |
| Private hospital | 1 (9) |
| **Months since previous TKR^b^** | |
| <3 | 3 (50) |
| 3<6 | 2 (33) |
| 6<12 | 1 (17) |
| **Body mass index (kg/m²)** | |
| 18.5<25 | 2 (22) |
| 25<30 | 1 (11) |
| 30<40 | 3 (33) |
| ≥40 | 3 (33) |
| **Ethnicity** | |
| White British | 7 (78) |
| Indian | 1 (11) |
| African Caribbean | 1 (11) |
| **Disability or health condition that could affect ability to use a website or carry out gentle exercises^c^** | |
| Dyslexia and dyspraxia | 1 (11) |
| Visual impairment | 2 (22) |
| Hand pain/swelling | 1 (11) |
| **Living location** | |
| Yorkshire and the Humber | 8 (89) |
| Scotland | 1 (11) |
| **Highest educational qualification** | |
| None | 2 (22) |
| Apprenticeship | 1 (11) |
| Vocational qualification (or equivalent) | 3 (33) |
| Undergraduate degree | 3 (33) |
| **Current employment status** | |
| Employed full-time | 1 (11) |
| Employed part-time | 1 (11) |
| Retired | 6 (67) |
| Medically disabled | 1 (11) |

^a^ Participants who were both awaiting and had undergone TKR were counted twice (11 TKRs in total).

^b^ Only includes participants who had previously undergone TKR (n=6).

^c^ Participants could report more than one option.

Abbreviations: NHS, National Health Service; Post, previously undergone TKR; Pre, listed for TKR; TKR, total knee replacement

# Table S16: Example quotes for issues identified with the Virtual Knee School prototype

| **VKS prototype section/aspect** | **Issues** | **Example quotes^a^** |
| --- | --- | --- |
| Design and overall content | Not realising it was possible to select the accordions (expandable headings). | “No, no, that [option to select the accordion] weren't obvious, no.” (Vera) |
|  | Feeling there was too much text. | "There’s a lot of information, a lot of reading, I’m more for flipping through as quick as I can as I’ve said, and there is a lot of information on the pages.” (Laurence) |
|  | Having difficulty locating and/or using the accessibility toolbar. | “Well, it [accessibility toolbar] is good for people who are very literate, fluent in computer and anything it’s alright, but I'm at the creeping stage. […] I’m still bottle fed.” (Zuri)^b^ |
|  | Being concerned about whether there was enough time to watch the videos. | “I didn’t know whether I would have enough time to watch the whole video.” (Haaniya) |
|  | Believing the patient stories were from real-life patients. | “I assumed, even with that [text] actually, that these were really people.” (Naomi)^b^ |
| Information architecture and navigation | Finding the tunnelling to the introductory section menu unhelpful/confusing. | “Well, I think when you open any website, it should take you to the homepage. […] Because that’s the starting point, the homepage is the starting point, the homepage tells you what the website’s, what the content of the website is.” (Glen) |
|  | Feeling overwhelmed by the volume of content due to the education dropdown menu displaying the titles of all 24 education pages/subpages. | “…too many categories there for me, I don’t know what…I mean, they might be all little tiny bits, I’ll go to the first one, let’s have a look about…” (Laurence) |
|  | Not realising it was possible to select the small triangles to display lower-level pages when using the meganav on a mobile device in portrait orientation. | “But then when I clicked on the arrow on information for your operation, it brought up the other menu. And then recovering. So and then it brings up the other menu. I didn’t realise that was there.” (Ella) |
|  | Feeling confused by the back and next buttons both going to the same page if the user accessed the last page in a section from the section menu. | “Well, first impression was it was a bit confusing because it’s basically taking you to the same place […]” (Glen) |
|  | Feeling extra hyperlinks would be useful for quickly checking other pages, and feeling confused about whether words in bold were hyperlinks. | “So if you click on those highlighted, presumably, can you get more about that, or is that just bold?” (Naomi)^b^ |
| Login section | Mistyping characters leading to the two passwords entered on the sign-up page not matching or the password entered on the login page being incorrect. | “These passwords do not match. […] On my phone I can press a button and I can actually read them. That’s what I’m used to.” (Arthur) |
| Main section | Feeling the main homepage did not make it clear that the website had three main sections. | “Well, what I will say is if I was on your homepage now, I personally, I can only speak personally, I don’t think it would be obvious to me that this website had beyond the homepage three key sections.” (Glen) |
|  | Feeling it should be clearer that the website provides information related to the peri- and post-operative phases, rather than just the pre-operative phase. | “Well, I think you should make it very clear that it’s covering every aspect, you know? Leading up to it, during and after.” (Glen) |
|  | Feeling the three homepage picture buttons did not indicate where to find the information the user wanted. | “But it’s…they’re not really telling me…like the knee joint, can I see a knee joint, it doesn’t tell me on those where I’m going to find it if you know what I mean.” (Laurence) |
|  | Feeling a link to the *‘Contact us’* page should be included in the website footer for consistency with other websites. | “Maybe the *‘Contact us’* as well could also be down here [in the website footer]. Because on most websites it’s usually at the bottom of the page.” (Ella) |
|  | Considering using the VKS email address to ask questions about the user’s own operation. | “I’m thinking I might ask something about my knee operation, possibly. Or if there was anything I couldn’t quite understand on the School, you know, on the actual pages.” (Arthur) |
| Introductory section | Feeling there was too much information on the introductory section menu. | "I think there’s a lot of information, like you can change the colour of the background. There’s a lot of information to process; for me I don’t think I would change the colour of the background but if other people wanted to, they could.” (Jessica) |
|  | Feeling confused by the instructions on how to use the website. | "I found it a little bit confusing, only for, there seemed to be a lot of information there, so, it’s hard to explain. Because it seemed to be kind of repeating itself a little bit over, on the, on the second one.” (Ella) |
|  | Finding the PDF of the Phase 1b modified Delphi study recommendations too detailed and *“very confusing”.* | “I think it's a lot of information. Maybe too much. I'm just, it's very confusing. Because to me this this ,like this section here for instance. I expected to be able to click on.” (Ella) |
|  | Feeling it would be helpful to amend the wording of certain answers on the *‘Common questions’* page. | “And you can say that it relieves stiffness as well. Because exercise definitely relieves stiffness.” (Haaniya) |
|  | Feeling it would be helpful to cover what to do if the user has bilateral knee problems on the *‘Common questions’* page. | “Now, whether that’s something that you can think about and, you know, have a line saying, somebody says I have, you know, I’ve got a problem with both knees but I’m having one of them done first sort of thing, does that change the way I approach anything?” (Glen) |
| Education section | Requesting further information about specific topics. | It’s leading me to ask more questions a bit now. I’m wanting…because they’re obviously going to do my second knee, one guy said about six weeks after the first one. How long…I would need to find out roughly how long it takes for the scar to heal enough to get back to exercising in the pool, that’s something I don’t know actually.” (Laurence) |
|  | Feeling the *‘Goal setting’* page should provide more encouragement for users who do not meet their goals. | “I think it should say to encourage the people who, even if you don’t meet your goals and you think you… not to be disappointed but to carry on at your own level and what you can achieve, rather than be upset with what you’ve not achieved, not to get disheartened.” (Haaniya) |
|  | Wanting post-operative goals to look forward to and *“something visual”.* | “So maybe if there's something visual there that would possibly put that thought into somebody's head, you know, look forward to this kind of goal.” (Ella) |
| Exercise section | Feeling confused about whether the exercise section was for the pre- or post-operative phase. | “It was just how I’d read it with the recovery afterwards. Sorry about the message that came up. Yeah, it's the way that it says it can manage your pain before your operation and recover faster afterwards. It was just my mind thinking it was for afterwards too.” (Ella) |
|  | Highlighting queries or concerns about specific aspects of the exercise section text. | “It [at least two exercise sessions a week] doesn’t sound enough really.” (Arthur) |
|  | Thinking the exercise category titles related to the videos above them rather than below them. | “But I think it’s the way I had it, because if you look, that’s category 1 and there’s no […] no break in between, is there?” (Jessica) |
|  | Missing the *‘Submit’* button on the goal-setting and review forms. | “I used to be observant when I was younger but I’m not as observant as I should be and I just didn’t spot the submit button.” (Glen) |
|  | Entering numbers in the goal-setting form as words rather than numerals. | “Oh, you’ve got to enter a number.” (Glen) |
|  | Finding it challenging to set appropriate exercise goals due to unfamiliarity with the VKS exercise programme. | “How many different exercises? Well, I only can walk, so it’s just one then, isn’t it? […] It will make a bit more sense after we’ve seen that [Virtual Knee School exercise plan].” (Jessica) |

^a^ The participants were interviewed in the following order: Ella, Jessica, Glen, Arthur, Vera, Haaniya, Laurence, Naomi, Zuri. There are a larger number of quotes from the first few participants because issues were identified and addressed on an iterative basis. Unless otherwise indicated, the quotes are from participants who used the VKS prototype prior to the change(s) detailed in Table 6 being made to address the issue.

^b^ Quote is from a participant who used the VKS prototype after the change(s) detailed in Table 6 were made to address the issue, suggesting the issue was not fully resolved.

Abbreviations: PDF, Portable Document Format; VKS, Virtual Knee School

# References

1. Anderson AM, Comer C, Smith TO, Drew BT, Pandit H, Antcliff D, et al. Consensus on pre-operative total knee replacement education and prehabilitation recommendations: a UK-based modified Delphi study. BMC Musculoskelet Disord. 2021;22(1):352.
